# Hydrodynamic analysis of the magnetic field dependent viscous fluid flow and thermosolutal convection between rotating channels

**DOI:** 10.1038/s41598-022-20959-1

**Published:** 2022-10-13

**Authors:** Aamir Khan, Muhammad Sohail Khan, Amjad Ali Pasha, Riadh Marzouki, Mustafa Mutiur Rahman, Omar Mahmoud, Ahmed M. Galal, S. A. Najati

**Affiliations:** 1 Department of Mathematics and Statistics, The University of Haripur KPK, Haripur, Pakistan; 2 School of Mathematical Sciences, Jiangsu University, Zhenjiang, 212013 Jiangsu China; 3 Faculty of Engineering, Aerospace Engineering Department, King Abdulaziz University, Jeddah, Saudi Arabia; 4 Chemistry Department College of Sciences, King Khalid University, Abha, 6141 Saudi Arabia; 5 Chemistry Department, Faculty of Sciences of Sfax, University of Sfax, Sfax, 3038 Tunisia; 6 Department of Mechanical and Mechatronics Engineering, University of Waterloo, Waterloo, ON N2L 3G1 Canada; 7 Petroleum Engineering, Faculty of Engineering and Technology, Future University in Egypt, New Cairo, 11835 Egypt; 8 Department of Mechanical Engineering, College of Engineering in Wadi Alddawasir, Prince Sattam bin Abdulaziz University, Al-Kharj, Saudi Arabia; 9 Production Engineering and Mechanical Design Department, Faculty of Engineering, Mansoura University, P.O 35516 Mansoura, Egypt; 10 Department of Mathematics and Statistics, College of Science, Taif University, P.O. Box 11099, Taif, 21944 Saudi Arabia

**Keywords:** Mathematics and computing, Physics

## Abstract

According to research, exposing a person to a magnetic field enhances blood flow and minimizes their risk of suffering a heart attack. Ferrohydrodynamics is the study of fluid motion mechanics that is affected by strong magnetic polarisation forces (FHD). Ferrofluids may transmit heat in a variety of ways by using magnetic fluids. This behaviour is demonstrated by liquid-cooled speakers, which utilise less ferrofluid to prevent heat from reaching the speaker coil. This modification boosts the coil’s ability to expand, which enables the loudspeaker to create high-fidelity sound. It is investigated how the fluid dynamics of spinning, squeezing plates are affected by thermosolutal convection and a magnetic field dependent (MFD) viscosity. Standard differential equations are used to represent the equations of the modified form of Navier Stokes, Maxwell’s, and thermosolutal convection. The magnetic field, modified velocity field equations, and thermosolutal convection equations all yield suitable answers. Additionally computed and thoroughly detailed are the MHD torque and fluid pressure that are imparted to the top plate. To create a technique with quick and certain convergence, the resulting equations for uniform plates are solved using the Homotopy Analysis Method (HAM) with appropriate starting estimates and auxiliary parameters. The validity and reliability of the HAM outcomes are shown by comparing the HAM solutions with the BVP4*c* numerical solver programme. It has been found that a magnetic Reynolds number lowers the temperature of the fluid as well as the tangential and axial components of the velocity field. Additionally, when the fluid’s MFD viscosity rises, the axial and azimuthal components of the magnetic field behave in opposition to one another. This study has applications in the development of new aircraft take-off gear, magnetorheological airbags for automobiles, heating and cooling systems, bio-prosthetics, and biosensor systems.

## Introduction

The study of the mechanics of fluid motion that is impacted by powerful magnetic polarisation effects is known as ferrohydrodynamics (FHD). Ferrofluids may transmit heat in a variety of ways by using magnetic fluids. This behaviour is demonstrated by liquid cooled speakers, which utilise less ferrofluid to prevent heat from reaching the speaker coil^1^. This modification boosts the coil’s ability to expand, which enables the loudspeaker to create high fidelity sound. A drop of ferrofluid going through the body can be used to deliver medications to a precise area in the human body using magnetic fluids^2^, which can be propelled by a magnetic field. Lenz’s law states that an electric current develops as it moves through a magnetic field, and the conductor also develops its own magnetic field. Since the currents are observed by the movement of the fluid passing through the magnetic field, the Lorentz force acts on the mass and increases the speed of the. In MHD, the field affects movement and vice versa. The concept is hence non-linear^3,4^. Researchers have shown that being exposed to a magnetic field enhances blood flow to the body and lowers the chance of a heart attack. In an experiment conducted by Rongjia Tao and Ke Huang of the University of Michigan and Temple University, respectively, it was discovered that a minute’s exposure to a magnetic field perpendicular to the direction of blood flow caused a small sample of blood in a tube viscera to compress blood viscosity by 33 to 4.75*cp*. Within 200 minutes of no more contact to the fluid, the viscosity may have grown noticeably to 5.4*cp*, which is still well within the acceptable range of 7*cp*.

A substance or metal cube made of two or more homogeneous or heterogeneous metals or non-metallic alloys is called an alloy. These alloys mixed mixes with unique characteristics or employed a heat transfer medium. Steel, brass, phosphor bronze, and solder are a few examples of alloys. Aerospace research, powder technology, hip joint replacement, and surgical implantation are just a few of the industries that employ alloys. The three types of alloys are alpha alloys, beta alloys, and alpha beta alloys. An industrially developed titanium alloy combining aluminium and titanium derivatives is called Alpha Alloys. Due to their changed heat transfer bulk, these alloys compromise the quality of the fuse bulk, and as a result, the alpha alloy is used more frequently in temperature application operations. Beta alloy is mostly utilised in cold rolling of sheets and welding systems. Elshekh et al.^5^ studied magnetic squeeze film flow from the magnetic field among rotating disk generated effects, considering as,

$$\begin{aligned} B_r = \frac{\delta r}{2D(1 - \delta t)}m'({\eta} ), B_\theta = \frac{rN_o}{\sqrt{1 - \delta t}}n({\eta} ), B_z = \frac{-\delta M_o}{\sqrt{1 - \delta t}}m({\eta} )\end{aligned}$$

with velocity components

$$\begin{aligned}u = \frac{\delta r}{2(1 - \delta t)}f'({\eta} ), v = \frac{\Omega r}{\sqrt{1 - \delta t}}g({\eta} )\end{aligned}$$

decrease the responsiveness of the radial and azimuthal magnetic fields and measure the response to the accompanying disc rotation for a value of the bachelor number. In the flow of the posterior portion of a vertical infinite plate in a rotating structure, Mutua et al.^6^ investigated the Stokes challenge. They came to the conclusion that any or all factors can affect the temperature and speed of a liquid. As a result, the rate of heat transmission along the axis and the skin’s resistance are both impacted. For both free cooling and heating in plate variations, the speed profile, magnetic parameters M, and Ec number EC enhance M convection. MHD was investigated by Seth et al.^7^ and shown to be amplified with rising values of time and Hall parameters, mass transfer, heat transfer in flow, and mass transfer with porous flat plates, but to be amplified with decreasing values of devolution parameters and magnetic field parameters. In order to maintain heat radiation, Victor^8^ employed the finite component approach to examine the volatile MHD free convection cutlet transfer inside the two vertical porous plates. Due to the radiation characteristics and the predental number, it was discovered that the temperature velocity was higher than the velocity. In other words, the fluid particle’s grashof numbers and magnetic properties were unaffected. The volatile MHD QUETT flow in two infinitely parallel porous plates was studied using the coefficient^9^ in a bent magnetic field with rate of heat transfer in temperature. The bottom plate was regarded as permanent and porous. He found that when the magnetic field grows, the fluid’s velocity decreases. In the presence of an externally applied magnetic field, Verma^10^ examined the lubrication of a magnetic fluid twisting film between two approaching surfaces. He assumed that the applied magnetic field *M* has the following components. He assumed that the applied magnetic field *M* has components of the form:

$$\begin{aligned} M_x = M(x)\cos \theta , \,\,\,M_y = M(x)\sin \theta ,\,\,\ M_z = 0\,\,\,\,\,\ \text {where}\,\,\,\ \theta = \theta (x,y).\end{aligned}$$

trying to squeeze flux with spinning disks is also examined in depth by^11–14^ under influences of changing magnetic fields. For the simulation of the magnetic film problem, Rashidi et al.^15^ used the Pade DTM hybrid method, which demonstrated good convergence consistency, and versatility. The objective of this research is to examine the existence of exponential heating decay and transversal magnetic field^16–18^ for the influence of different electrical conductivity on a free convection flow of a viscous electrically conducive fluid and heat transfer through an isothermal non conductive vertical plat. The heat transfer and aerodynamic forces rate may be conveniently adjusted by applying an appropriate magnetic field. Morley examine the impact of MHD given in^19^ known as blanket. The blanket is placed among both magnetic field and plasma’s spindles, absorbing neutrons that transform its energies into heat, which is subsequently taken away by an appropriate cooling, restricting neutrons from entering the magnets and therefore preventing radiation destruction. For drag reduction several fascinating improvements have been presented in the domains of MHD propulsion and remote energy deposition. The flow is represented by the system of nonlinear equations which includes fundamental ohmic law, Maxwell, momentum and energy equations^24–27^. The system of nonlinear equations given in^28^ was solved by finite difference technique. Flow of non-Newtonian electrically conducting fluid is an essential phenomena, since we are dealing with fluid flow, which displays various patterns in the effect of magnetic forces in most real circumstances. In such situations, the feature of the flow MHD must be taken into account. Siddiqui et al.^29^ utilizing homotopy perturbation method to obtain solutions for squeezed MHD two dimensional flow among parallel plates. Newtonian nonisothermal fluid flow among unsteady squeezed permeable disks for changeable magnetic-field will be investigate. The PDEs well be transform into nonlinear coupled ODEs using similarity transformations. The formed nonlinear DEs describing the flow characteristics within the geometry under observation will also analyze through numerical and analytical approaches. Analyses will also be conducted amongst the solutions. The convergence of results will be explored as well. Flow pattern will be explained graphically^30–32^ under the impact of non-dimensional factors. Nazeer et al.^33^ stadied the thermal transport of two-phase physiological flow of non Newtonian fluid through an inclined channel with flexible walls. Yassen et al.^34^, investigated the theoretical study of transport of MHD peristaltic flow of fluid under the impact of viscous dissipation. Nazeer et al.^35^ investigated the heat transmission in a magnetohydrodynamic multiphase flow induced by metachronal propulsion through porous media with thermal radiation. Zubaidi et al.^36^ studied the numerical study of squeezing flow past a Riga plate with activation energy and chemical reactions: effects of convective and second-order slip boundary conditions. Quraishi et al.^37^ the influence of radially magnetic field properties in a peristaltic flow with internal heat generation: Numerical treatment. Awan et al.^38^ examined the numerical treatments to analyze the nonlinear radiative heat transfer in MHD nanofluid flow with solar energy. Raja et al.^39^ studied the integrated intelligent computing application for effectiveness of Au nanoparticles coated over MWCNTs with velocity slip in curved channel peristaltic flow. Awan et al.^40^ examined the backpropagated intelligent computing networks for 3*D* nanofluid rheology with generalized heat flux. Majeed et al.^41^ studied the mathematical analysis of MHD CNTs of rotating nanofluid flow over a permeable stretching surface. Shahzad et al.^42^ investigated the effects of magnetohydrodynamics flow on multilayer coatings of Newtonian and non Newtonian fluids through porous inclined rotating channel. Zeeshan et al., examined the radiative bioconvection nanofluid squeezing flow between rotating circular plates: Semi-numerical study with the DTM Padé approach.

This demonstrates the aforementioned heavy emphasis on fluid motion in a magnetic field with high rotational velocity in an MFD viscosity. MFD thermosolutal convection also has applications in various fields such as aerospace-technology, powder technologies, liquid cooled speaker, pump building, auto magneto rheological suspension system, new aviation system landing gears, biological prosthetics, hip joints replacement processes and the surgical implantation procedure. Due to its essential scientific and practical importance, it is sought to characterize the impact of rotation and how MFD thermosolutal convection, MFD viscosity impacts magnetization of the fluids. The information available at the theme revealed that the squeezing-flow MFD viscosity between rotating plates for a viscous fluid under the MFD thermosalutal and externally applied magnetic field in Cartesian coordinates was never reported and is the very first study to carried out in the literature. Hence, the suggestework is the best approach toward such problems and is a way of motivation for researchers bringing a new idea of studying the flow between unsteady rotating parallel plates. In the following sections, the problem is studied, examined and explained using tables and graphs.

## Formulation of the problem

In this article the viscous fluid is suppose to be axisymmetric and uncompressed, squeezed between parallel plates, distanced by a length of $D(t) = l\sqrt{1 - \alpha t}$, in which *l* represents a plate separation at $t = 0$^20,22,23^. The two plates being squeezed unless they meet at $t = \frac{1}{\alpha }$ for $\alpha > 0$, and they are separated for $\alpha < 0$. Both plates rotate with a distinct angular velocities of $\frac{\Omega _{l}}{1-\alpha t}$ and $\frac{\Omega _{u}}{1-\alpha t}$, where $\Omega _{l}$, $\Omega _{u}$ representing the angular speeds of bottom and top plates correspondingly^20–22^. The bottom plate is fixed, while the top plate moves near or apart from the bottom plate. As compared to magnetic forces, Electrical forces are significantly less and thus neglected in the current research problem. Applied magnetic field $(B_{x}, B_{y}, B_{z})$ inside the fluid generates the induced magnetic field $(B_{x}, B_{y}, B_{z})$ which can be written as:

$$\begin{aligned} H_{x} = \frac{\alpha xM_{o}}{\mu _{2}(1 - \alpha t)},\,\,\,\,H_{y} = \frac{xN_{o}}{\mu _{2}(1 - \alpha t)}, \,\,\, H_{z} = \frac{-xM_{o}}{\mu _{1}(1 - \alpha t)^{0.5}} \end{aligned}$$

Wherever the magnetic permeability of the external and the inner media among these plates is $N_{o}$ and $M_{o}$, $H_{x}$, $H_{y}$, $H_{z}$ and $\mu _{1}$, $\mu _{2}$. On the bottom plate^5^ the aforementioned magnetic field parameters are zero. Incompressible fluids is considered, with a variable viscosity provided by $\mu = \mu _{o}( 1 + \delta .B )$ where $\mu _{o}$, represents the fluid’s viscosity in the absence of a magnetic-field^25^. The alteration viscosity-coefficient $\delta$ are assumed to be isotropic, i.e. $\delta _{x}$ = $\delta _{y}$ = $\delta _{z}$. Hence, $\mu$ in the component form can be written as $\mu _{x} = \mu _{o}( 1 + \delta B_{x} )$, $\mu _{y} = \mu _{o}( 1 + \delta B_{y} )$ and $\mu _{z} = \mu _{o}( 1 + \delta B_{z} )$. Furthermore, *M*, *B*, and *H* may linked by the equation $B = \mu _{p}( M + H )$, in which *M* is the magnetization when the magnetic field is *H* and the vacuum magnetic-permeability $\mu _{p}$. The influence of shearing dependency on viscosity is not examined because it has a minor influence on a wide spin and high field monodispersive system. A linear change of magnet viscosity was initially employed as a small field variation, thus $B = \mu _{p}( M_{o} + H_{o} )$ where $H_{o}$ is uniform magnetic-field and $M_{o}$ is the magnetization when the magnetic field is $H_{o}$. The equations which governing the flow and heat/mass transfers in viscous fluid are:

Continuity equation:

1 $$\begin{aligned} \dfrac{\partial u}{\partial x}+\dfrac{\partial v}{\partial y}+\dfrac{\partial w}{\partial z}=0, \end{aligned}$$

$x-component$ of Navier Stokes equation:

2 $$\begin{aligned} \begin{array}{c} \rho \left[ \frac{\partial u }{\partial t} + u\frac{\partial u }{\partial x} + v\frac{\partial u }{\partial y } + w \frac{\partial u }{\partial z} \right] =-\frac{\partial P }{\partial x} + \mu \left[ \frac{\partial ^2 u }{\partial x^2} +\frac{\partial ^2 u }{\partial y^2 }+\frac{\partial ^2 u }{\partial z^2} \right] +\\ \frac{1}{\mu _2} \left[ B_z\frac{\partial B_x}{\partial z}-B_z\frac{\partial B_z}{\partial x}-B_y\frac{\partial B_y}{\partial x}+B_y\frac{\partial B_x}{\partial y} \right] , \end{array} \end{aligned}$$

$y-component$ of Navier Stokes equation:

3 $$\begin{aligned} \begin{array}{c} \rho \left[ \frac{\partial v }{\partial t}+u\frac{\partial v }{\partial x}+v\frac{\partial v }{ \partial y }+w\frac{\partial v }{\partial z} \right] =-\frac{\partial p }{\partial y } +\mu \left[ \frac{\partial ^2 v }{\partial x^2} +\frac{\partial ^2 v }{\partial y^2 }+\frac{\partial ^2 v }{\partial z^2} \right] + \\ \frac{1}{\mu _2} \left[ B_x\frac{\partial B_y}{\partial x}-B_x\frac{ \partial B_x}{\partial y}-B_z\frac{\partial B_z}{\partial y}+B_z\frac{\partial B_y}{\partial z} \right] , \end{array} \end{aligned}$$

$z-component$ of Navier Stokes equation:

4 $$\begin{aligned} \begin{array}{c} \rho \left[ \dfrac{\partial w }{\partial t}+u\dfrac{\partial w }{\partial x}+v\dfrac{\partial w }{ \partial y }+w\dfrac{\partial w }{\partial z} \right] =-\dfrac{\partial p }{\partial z } +\mu \left[ \dfrac{\partial ^2 w }{\partial x^2} +\dfrac{\partial ^2 w }{\partial y^2 }+\dfrac{\partial ^2 w }{\partial z^2} \right] +\\ \dfrac{1}{\mu _2} \left[ B_y\dfrac{\partial B_z}{\partial y}-B_y\dfrac{ \partial B_y}{\partial z}-B_x\dfrac{\partial B_x}{\partial z}+B_x\dfrac{\partial B_z}{\partial x} \right] , \end{array} \end{aligned}$$

$x-component$ of Magnetic field equation :

5 $$\begin{aligned} \begin{array}{c} \dfrac{\partial B_x}{\partial t}= \left[ u\dfrac{\partial B_y}{\partial y}+B_y\dfrac{\partial u}{\partial y}-v\dfrac{\partial B_x}{\partial y}-B_x\dfrac{\partial v}{\partial y}-w\dfrac{\partial B_x}{\partial z}-B_x\dfrac{\partial w}{\partial z}+ u\dfrac{\partial B_z}{\partial z}+B_z\dfrac{\partial u}{\partial z}\right] +\\ \dfrac{1}{\delta \mu _2} \left[ \dfrac{\partial ^2 B_x}{\partial x^2}+\dfrac{\partial ^2 B_x}{\partial y^2}+\dfrac{\partial ^2 B_x}{\partial z^2} \right] , \end{array} \end{aligned}$$

$y-component$ of Magnetic field equation:

6 $$\begin{aligned} \begin{array}{c} \dfrac{\partial B_y}{\partial t}= \left[ v\dfrac{\partial B_z}{\partial z}+B_z\dfrac{\partial v}{\partial z}-w\dfrac{\partial B_y}{\partial z}-B_y\dfrac{\partial w}{\partial z}-u\dfrac{\partial B_y}{\partial x}-B_y\dfrac{\partial u}{\partial x}+ v\dfrac{\partial B_x}{\partial x}+B_x\dfrac{\partial v}{\partial x}\right] +\\ \dfrac{1}{\delta \mu _2} \left[ \dfrac{\partial ^2 B_y}{\partial x^2}+\dfrac{\partial ^2 B_y}{\partial y^2}+\dfrac{\partial ^2 B_y}{\partial z^2} \right] , \end{array} \end{aligned}$$

$z-component$ of Magnetic field equation:

7 $$\begin{aligned} \begin{array}{c} \dfrac{\partial B_z}{\partial t}= \left[ w\dfrac{\partial B_x}{\partial x}+B_x\dfrac{\partial w}{\partial x}-u\dfrac{\partial B_z}{\partial x}-B_z\dfrac{\partial u}{\partial x}-v\dfrac{\partial B_z}{\partial y}-B_z\dfrac{\partial v}{\partial y}+ w\dfrac{\partial B_y}{\partial y}+B_y\dfrac{\partial w}{\partial y}\right] +\\ \dfrac{1}{\delta \mu _2} \left[ \dfrac{\partial ^2 B_z}{\partial x^2}+\dfrac{\partial ^2 B_z}{\partial y^2}+\dfrac{\partial ^2 B_z}{\partial z^2} \right] , \end{array} \end{aligned}$$

Energy equation:^25^

8 $$\begin{aligned} \begin{array}{c} \bigg [\rho _{o}C_{V,H} - \mu _{o}H.\bigg (\frac{\partial M}{\partial T}\bigg )_{V,H}\bigg ]\left[ \dfrac{\partial T}{\partial t}+u\dfrac{\partial T}{\partial x}+v\dfrac{\partial T}{\partial y}+w\dfrac{\partial T}{\partial z} \right] = K_{1}\left[ \dfrac{\partial ^2 T}{\partial x^2}+\dfrac{\partial ^2 T}{\partial y^2}+\dfrac{\partial ^2 T}{\partial z^2}\right] - \\ \mu _{o}T\bigg (\frac{\partial M}{\partial T}\bigg ).\left[ \dfrac{\partial T}{\partial t}+u\dfrac{\partial T}{\partial x}+v\dfrac{\partial T}{\partial y}+w\dfrac{\partial T}{\partial z} \right] , \end{array}{c} \end{aligned}$$

Concentration equation:^25^

9 $$\begin{aligned} \begin{array}{c} \bigg [\rho _{o}C_{V,H} - \mu _{o}H.\bigg (\frac{\partial M}{\partial C}\bigg )_{V,H}\bigg ]\left[ \dfrac{\partial C}{\partial t}+u\dfrac{\partial C}{\partial x}+v\dfrac{\partial C}{\partial y}+w\dfrac{\partial V}{\partial z} \right] = K^{'}_{1}\left[ \dfrac{\partial ^2 C}{\partial x^2}+\dfrac{\partial ^2 C}{\partial y^2}+\dfrac{\partial ^2 C}{\partial z^2}\right] - \\ \mu _{o}C\bigg (\frac{\partial M}{\partial C}\bigg ).\left[ \dfrac{\partial C}{\partial t}+u\dfrac{\partial C}{\partial x}+v\dfrac{\partial C}{\partial y}+w\dfrac{\partial C}{\partial z} \right] . \end{array}{c} \end{aligned}$$

where $\varrho$ is the electrical conductivity, $C_{V,H}$ is specific heat at constant volume and magnetic field, *T* is fluid temperature, *C* is solute concentration, *M* is magnetization, thermal conductivity, $K_{1}$, $K^{'}_{1}$ are solute conductivity, $\rho _{o}$ is reference density *p* is pressure, $\mu$ is variable dynamic viscosity and *U* is the velocity of the fluid.

## Boundary conditions

The boundary conditions are chosen as:

10 $$\begin{aligned} \begin{aligned}{}&v = \frac{\Omega _{l}}{1-\alpha t}, u = w = B_{r} = B_{\theta } = B_{z} = 0,T = T_{l}, C = C_{l}\,\, at\,\, z = 0, \\&v = \frac{\Omega _{u}r}{1-\alpha t}, w = \frac{dD(t)}{dt},u = B_{r} = 0, B_\theta = \frac{rN_{o}}{1-\alpha t}, B_z = \frac{-rM_{o}}{\sqrt{1-\alpha t}}, \\&T = T_{u}, C = C_{u} \,\,at\,\, z = D(t). \end{aligned} \end{aligned}$$

where $\alpha$ is the thermal diffusivity, the specific heat at constant pressure is $c_p$, *u*, *v* and *w* are respectively the radial, azimuthal and axial components of the velocity field, *D* is diffusion coefficient, $T_{l}$, $C_{l}$, $T_{u}$ and $C_{u}$ are temperature and concentration at lower and upper plate respectively.

Due to the non-linearity factor and selection of the boundary conditions for the set of coupled equations (1–10), it is too complicated to solve it in the form of partial differential equations (PDEs). To solve this issue, the set of PDEs have been converted into the set of ordinary differential equations (ODEs) by introducing the Lie group of similarity transformations^26^. These similarity transformations are chosen as, $u=\dfrac{\beta x}{(1-\beta t)}f'({\eta} )$,      $v=\dfrac{\Omega _l x}{(1-\beta t)}g({\eta} )$,      $w=\dfrac{-\beta l}{(1-\beta t)^{1/2}}f({\eta} )$,      $B_x=\dfrac{\beta x M_0}{l(1-\beta t)}h'({\eta} )$,      $B_y=\dfrac{xN_0}{(1-\beta t)}k({\eta} )$,      $B_z=\dfrac{-\beta M_0}{(1-\beta t)^{1/2}}h({\eta} )$,      $\theta =\dfrac{T-T_u}{T_l-T_u}$,      $\Psi =\dfrac{C-C_u}{C_l-C_u}$,      $\eta =\dfrac{z}{l(1-\beta t)^{1/2}}$.

Equation (1) is identically satisfied and Eqs. (2–9) takes the following form

11 $$\begin{aligned} \xi _{vis} \xi ^{2}_{sq}f'''' - \xi ^{3}_{sq}\bigg [ 3f'' + \eta f''' - 2f\,f''' + 2\xi ^{2}_{z}\xi _{mag}\bigg ({\eta} hh''&+ hh' + 2h^{2}f'' - 2fhh''\bigg )\bigg ] \nonumber \\ \quad\quad \quad\quad+ 2\xi ^{2}_{rot}\bigg (gg' - \xi ^{2}_{\theta }kk'\bigg ) = 0, \end{aligned}$$

12 $$\begin{aligned} \xi _{vis} g'' - \xi _{sq}\bigg ( 2g + \eta g' + 2gf' - 2fg' \bigg ) + 2\xi _{z}\xi _{\theta }\bigg ( hk' - kh'\bigg ) = 0, \end{aligned}$$

13 $$\begin{aligned} h'' - \xi _{mag}\bigg ( h + \eta h' - 2fh' + 2hf' \bigg ) = 0, \end{aligned}$$

14 $$\begin{aligned} \xi _{\theta }k'' - \xi _{\theta }\xi _{mag}\bigg ( 2k + \eta k' - 2fk' \bigg ) - 2\xi _{z}hg' = 0, \end{aligned}$$

15 $$\begin{aligned} \theta ^{''} + \xi _{sq}\xi _{pr}\bigg (2f\theta ^{'} - \eta \theta ^{'}\bigg ) - \xi _{ec}\xi _{hm}\xi _{pm}\bigg ({\eta} h^{'} + h - 2fh^{'}\bigg )= 0, \end{aligned}$$

16 $$\begin{aligned} \Psi ^{''} + \xi _{sq}\xi _{pr}\bigg (2f\Psi ^{'} - \eta \Psi ^{'}\bigg ) - \xi _{ec}\xi _{hm}\xi _{sm}\bigg ({\eta} h^{'} + h - 2fh^{'}\bigg )= 0, \end{aligned}$$

and the boundary conditions are reduced to

17 $$\begin{aligned} \begin{aligned}{}&f(0) = f'(0) = h(0) = k(0) = 0, \Psi (0) = g(0) = \theta (0) = 1, \\&f(1) = 1/2, ,g(1) = \frac{\Omega {u}}{\Omega _{l}} = S, \theta (1) = \Psi (1) = f'(1) = 0, h(1) = k(1) = 1. \end{aligned} \end{aligned}$$

where $\xi _{ec} = \frac{\nu ^2\alpha }{2K_{1}(T_{l} - T_{u})\sqrt{1-\alpha t}}$ is Eckert number, $\xi _{z} = \frac{M_o}{l\sqrt{\mu _2\rho }}$ is strength of magnetic field in *z* direction, $\xi _{\theta } = \frac{N_o}{\Omega _{l}\sqrt{\mu _2\rho }}$ is strength of magnetic field in $\theta$ direction, $\xi _{sq} = \frac{\alpha l^2}{2\nu }$ is squeeze Reynolds number, $\xi _{pm} = -\frac{\partial M}{\partial T}$ is Pyromagnetic coefficient and $\xi _{sm} = -\frac{\partial M}{\partial C}$ is the salinity magnetic coefficient, $\xi _{rot} = \frac{\Omega _{l} l^2}{\nu }$ is rotational Reynolds number, $\xi _{bt} = \varrho \mu _{2}\nu$ is Bachelor number, $\xi _{mag} = \xi _{sq}\xi _{bt}$ is magnetic Reynolds number, $S = \frac{\Omega _{u}}{\Omega _{l}}$ is the relative angular velocity of discs, $\xi _{vis} = 1 + \delta _{v}\mu _{p}(M_{o} + H_{o})$ is magnetic field dependent viscosity parameter, $\xi _{pr} = \frac{\nu \rho C1}{K_{1}}$ is Prandtl number, $\xi _{hm} = \frac{\alpha M_{o}l}{\nu }$ is Hartmann number, .

## Approximate analytical solution

The analytic method HAM is used to solve system of Eqs. (9–15). Due to HAM, the functions $f({\eta} )$, $g({\eta} )$, $h({\eta} )$, $k({\eta} )$, $\theta ({\eta} )$ and $\Psi ({\eta} )$ can be expressed, by a set of base functions $\eta ^{c},\,\, c \ge 0$ as:^43^

18 $$\begin{aligned} f_{m}({\eta} )= \,\,& {} \sum _{\zeta =0}^{\infty } a_{\zeta }\eta ^{\zeta }, \end{aligned}$$

19 $$\begin{aligned} g_{m}({\eta} )= \,\,& {} \sum _{\zeta =0}^{\infty } b_{\zeta }\eta ^{\zeta }, \end{aligned}$$

20 $$\begin{aligned} \xi _{hm}({\eta} )= \,\,& {} \sum _{\zeta =0}^{\infty } c_{\zeta }\eta ^{\zeta }, \end{aligned}$$

21 $$\begin{aligned} k_{m}({\eta} )= \,\,& {} \sum _{\zeta =0}^{\infty } d_{\zeta }\eta ^{\zeta }, \end{aligned}$$

22 $$\begin{aligned} \theta _{m}({\eta} )= \,\,& {} \sum _{\zeta =0}^{\infty } e_{\zeta }\eta ^{\zeta }, \end{aligned}$$

23 $$\begin{aligned} \Psi _{m}({\eta} )= \,\,& {} \sum _{\zeta =0}^{\infty } f_{\zeta }\eta ^{\zeta }, \end{aligned}$$

where $a_{\zeta }$, $b_{\zeta }$, $c_{\zeta }$, $d_{\zeta }$, $e_{\zeta }$ and $f_{\zeta }$ are the constant coefficients to be determined. Initial approximations are chosen follows:

24 $$\begin{aligned} f_{0}({\eta} )= \,\,& {} (2A - 1)\eta ^3 - \frac{3}{2}(2A -1)\eta ^2 + A, \end{aligned}$$

25 $$\begin{aligned} g_{0}({\eta} )= \,\,& {} \Omega \eta , \end{aligned}$$

26 $$\begin{aligned} h_{0}({\eta} )= \,\,& {} \eta , \end{aligned}$$

27 $$\begin{aligned} k_{0}({\eta} )=\,\, & {} \eta , \end{aligned}$$

28 $$\begin{aligned} \theta _{0}({\eta} )= \,\,& {} 1 - \eta , \end{aligned}$$

29 $$\begin{aligned} \Psi _{0}({\eta} )=\,\, & {} 1 - \eta . \end{aligned}$$

The auxiliary operators are chosen as

30 $$\begin{aligned} {\ell }_{f} = \frac{\partial ^{4}}{\partial \eta ^{4}}, \,\,{\ell }_{g} = \frac{\partial ^{2}}{\partial \eta ^{2}}, \,\,{\ell }_{h} = \frac{\partial ^{2}}{\partial \eta ^{2}}, \,\,{\ell }_{k} = \frac{\partial ^{2}}{\partial \eta ^{2}},\,\, {\ell }_{\theta } = \frac{\partial ^{2}}{\partial \eta ^{2}}, \,\,{\ell }_{\Psi } = \frac{\partial ^{2}}{\partial \eta ^{2}}, \end{aligned}$$

with the following properties

31 $$\begin{aligned} {\ell }_{f} \left( \zeta _{1}\eta ^3 + \zeta _{2}\eta ^2 + \zeta _{3}\eta + \zeta _{4}\right)= \,\,& {} 0, \end{aligned}$$

32 $$\begin{aligned} {\ell }_{g}( \zeta _{5}\eta + \zeta _{6})= \,\,& {} 0, \end{aligned}$$

33 $$\begin{aligned} {\ell }_{h}( \zeta _{7}\eta + \zeta _{8})= \,\,& {} 0, \end{aligned}$$

34 $$\begin{aligned} {\ell }_{k}( \zeta _{9}\eta + \zeta _{10})= \,\,& {} 0, \end{aligned}$$

35 $$\begin{aligned} {\ell }_{\theta }( \zeta _{11}\eta + \zeta _{12})= \,\,& {} 0, \end{aligned}$$

36 $$\begin{aligned} {\ell }_{\Psi }( \zeta _{13}\eta + \zeta _{14})= \,\,& {} 0, \end{aligned}$$

where $\zeta _{1}$, $\zeta _{2}$, $\zeta _{3}$, $\zeta _{4}$, $\zeta _{5}$, $\zeta _{6}$, $\zeta _{7}$, $\zeta _{8}$, $\zeta _{9}$, $\zeta _{10}$, $\zeta _{11}$, $\zeta _{12}$, $\zeta _{13}$ and $\zeta _{14}$ are arbitrary constants.

The *Zeroth* order deformation problems can be obtained as:

37 $$\begin{aligned} (1 ;\beta ){\ell }_{f} \left[ {\bar{f}}({\eta} ;\beta ) - f_{0}({\eta} )\right]= \,\,& {} q\hbar _{f}{} \textit{N}_{f} \left[ {\bar{f}}({\eta} ;\beta ), {\bar{g}}({\eta} ;\beta ), {\bar{h}}({\eta} ;\beta ), {\bar{k}}({\eta} ;\beta )\right] , \end{aligned}$$

38 $$\begin{aligned} (1 ;\beta ){\ell }_{g} \left[ {\bar{g}}({\eta} ;\beta ) - g_{0}({\eta} )\right]= \,\,& {} q\hbar _{g}{} \textit{N}_{g} \left[ {\bar{f}}({\eta} ;\beta ), {\bar{g}}({\eta} ;\beta ), {\bar{h}}({\eta} ;\beta ), {\bar{k}}({\eta} ;\beta )\right] , \end{aligned}$$

39 $$\begin{aligned} (1 ;\beta ){\ell }_{h} \left[ {\bar{h}}({\eta} ;\beta ) - h_{0}({\eta} )\right]= \,\,& {} q\hbar _{h}{} \textit{N}_{h} \left[ {\bar{f}}({\eta} ;\beta ), {\bar{h}}({\eta} ;\beta ), {\bar{k}}({\eta} ;\beta )\right] , \end{aligned}$$

40 $$\begin{aligned} (1 ;\beta ){\ell }_{k} \left[ {\bar{k}}({\eta} ;\beta ) - k_{0}({\eta} )\right]= \,\,& {} q\hbar _{k}{} \textit{N}_{k} \left[ {\bar{f}}({\eta} ;\beta ), {\bar{h}}({\eta} ;\beta ), {\bar{h}}({\eta} ;\beta )\right] , \end{aligned}$$

41 $$\begin{aligned} (1 ;\beta ){\ell }_{\theta } \left[ {\bar{\theta }}({\eta} ;\beta ) - \theta _{0}({\eta} )\right]= \,\,& {} q\hbar _{\theta }{} \textit{N}_{\theta } \left[ {\bar{f}}({\eta} ;\beta ), {\bar{\theta }}({\eta} ;\beta ), {\bar{h}}({\eta} ;\beta )\right] , \end{aligned}$$

42 $$\begin{aligned} (1 ;\beta ){\ell }_{\Psi } \left[ {\bar{\Psi }}({\eta} ;\beta ) - \Psi _{0}({\eta} )\right]= \,\,& {} q\hbar _{\Psi }{} \textit{N}_{\Psi } \left[ {\bar{f}}({\eta} ;\beta ), {\bar{\theta }}({\eta} ; q), {\bar{h}}({\eta} ;\beta )\right] . \end{aligned}$$

The nonlinear operators of Eqs. (9–14) are defined as

43 $$\begin{aligned} \textit{N}_{f}\left[ {\bar{f}}({\eta} ;\beta ), {\bar{g}}({\eta} ;\beta ), {\bar{h}}({\eta} ;\beta ), {\bar{k}}({\eta} ;\beta )\right]= \,\,& {} \xi ^{2}_{sq}\frac{\partial ^4{\bar{f}}({\eta} ;\beta )}{\partial \eta ^4} - \xi ^{3}_{sq}\bigg [\eta \frac{\partial ^3{\bar{f}}({\eta} ;\beta )}{\partial \eta ^3} + 3\frac{\partial ^2{\bar{f}}({\eta} ;\beta )}{\partial \eta ^2} - 2f\frac{\partial ^3{\bar{f}}({\eta} ;\beta )}{\partial \eta ^3} \nonumber \\&+\,\, 2\aleph ^2_{c}\xi _{mag}\bigg ({\eta} {\bar{h}}({\eta} ;\beta )\frac{\partial ^2{\bar{h}}({\eta} ;\beta )}{\partial \eta ^2} + {\bar{h}}({\eta} ;\beta )\frac{\partial {\bar{h}}({\eta} ;\beta )}{\partial \eta } \nonumber \\&+\,\, 2\bar{h^2}({\eta} ;\beta )\frac{\partial ^2{\bar{f}}({\eta} ;\beta )}{\partial \eta ^2} - 2{\bar{h}}({\eta} ;\beta ){\bar{f}}({\eta} ;\beta )\frac{\partial ^2{\bar{h}}({\eta} ;\beta )}{\partial \eta ^2}\bigg )\bigg ] \nonumber \\&+\,\, 2\aleph ^2_{a}\bigg ({\bar{g}}({\eta} ;\beta )\frac{\partial {\bar{g}}({\eta} ;\beta )}{\partial \eta } - \aleph ^2_{d}{\bar{k}}({\eta} ;\beta )\frac{\partial {\bar{k}}({\eta} ;\beta )}{\partial \eta }\bigg ), \end{aligned}$$

44 $$\begin{aligned} \textit{N}_{g}[{\bar{f}}({\eta} ;\beta ), {\bar{g}}({\eta} ;\beta ), {\bar{h}}({\eta} ;\beta ), {\bar{k}}({\eta} ;\beta )]= \,\,& {} \frac{\partial ^2{\bar{g}}({\eta} ;\beta )}{\partial \eta ^2} - \aleph _b\bigg [2{\bar{g}}({\eta} ;\beta ) + \eta \frac{\partial {\bar{g}}({\eta} ;\beta )}{\partial \eta } + 2{\bar{g}}({\eta} ;\beta )\frac{\partial {\bar{f}}({\eta} ;\beta )}{\partial \eta } \nonumber \\&- \,\,2{\bar{f}}({\eta} ;\beta )\frac{\partial {\bar{g}}({\eta} ;\beta )}{\partial \eta } + 2\xi _{z}\xi _{\theta }\bigg ( h\frac{\partial {\bar{k}}({\eta} ;\beta )}{\partial \eta } - {\bar{k}}({\eta} ;\beta )\frac{\partial {\bar{h}}({\eta} ;\beta )}{\partial \eta }\bigg )\bigg ], \end{aligned}$$

45 $$\begin{aligned} \textit{N}_{h}[{\bar{f}}({\eta} ;\beta ), {\bar{h}}({\eta} ;\beta ), {\bar{k}}({\eta} ;\beta )]= \,\,& {} \frac{\partial ^2{\bar{h}}({\eta} ;\beta )}{\partial \eta ^2} - \xi _{mag}\bigg ({\bar{h}}({\eta} ;\beta ) + \eta \frac{\partial {\bar{h}}({\eta} ;\beta )}{\partial \eta } - 2{\bar{f}}({\eta} ;\beta )\frac{\partial {\bar{h}}({\eta} ;\beta )}{\partial \eta } \nonumber \\&+\,\, 2{\bar{h}}({\eta} ;\beta )\frac{\partial {\bar{f}}({\eta} ;\beta )}{\partial \eta }\bigg ), \end{aligned}$$

46 $$\begin{aligned} \textit{N}_{k}\left[ {\bar{f}}({\eta} ;\beta ), {\bar{h}}({\eta} ;\beta ), {\bar{k}}({\eta} ;\beta )\right]= \,\,& {} \xi _{\theta }\frac{\partial ^2{\bar{k}}({\eta} ;\beta )}{\partial \eta ^2} - \xi _{\theta } \xi _{mag}\bigg [ (2{\bar{k}}({\eta} ;\beta ) + \eta \frac{\partial {\bar{k}}({\eta} ;\beta )}{\partial \eta } - 2f\frac{\partial {\bar{k}}({\eta} ;\beta )}{\partial \eta }\bigg ) \nonumber \\&- \,\,2\xi _{z}{\bar{h}}({\eta} ;\beta )\frac{\partial {\bar{g}}({\eta} ;\beta )}{\partial \eta }, \end{aligned}$$

47 $$\begin{aligned} \textit{N}_{\theta }\left[ {\bar{f}}({\eta} ;\beta ), {\bar{\theta }}({\eta} ;\beta ), {\bar{h}}({\eta} ;\beta )\right]= \,\,& {} \frac{\partial ^{2}{\bar{\theta }}({\eta} ;\beta )}{\partial \eta ^2} + \xi _{sq}\xi _{pr}\bigg (2{\bar{f}}({\eta} ;\beta )\frac{\partial {\bar{\theta }}({\eta} ;\beta )}{\partial \eta } - \eta \frac{\partial {\bar{\theta }}({\eta} ;\beta )}{\partial \eta }\bigg ) \nonumber \\&-\,\, \xi _{ec}\xi _{hm}\xi _{pm}\bigg ({\eta} \frac{\partial {\bar{h}}({\eta} ;\beta )}{\partial \eta } + {\bar{h}}({\eta} ;\beta ) - 2{\bar{f}}({\eta} ;\beta )\frac{\partial {\bar{h}}({\eta} ;\beta )}{\partial \eta } \bigg ), \end{aligned}$$

48 $$\begin{aligned} \textit{N}_{\Psi }\left[ {\bar{f}}({\eta} ;\beta ), {\bar{\Psi }}({\eta} ;\beta ), {\bar{h}}({\eta} ;\beta )\right]= \,\,& {} \frac{\partial ^{2}{\bar{\Psi }}({\eta} ;\beta )}{\partial \eta ^2} + \xi _{sq}\xi _{pr}\bigg (2{\bar{f}}({\eta} ;\beta )\frac{\partial {\bar{\Psi }}({\eta} ;\beta )}{\partial \eta } - \eta \frac{\partial {\bar{\Psi }}({\eta} ;\beta )}{\partial \eta }\bigg ) \nonumber \\&-\,\, \xi _{ec}\xi _{hm}\xi _{sm}\bigg ({\eta} \frac{\partial {\bar{h}}({\eta} ;\beta )}{\partial \eta } + {\bar{h}}({\eta} ;\beta ) - 2{\bar{f}}({\eta} ;\beta )\frac{\partial {\bar{h}}({\eta} ;\beta )}{\partial \eta } \bigg ), \end{aligned}$$

where $\beta$ is an embedding parameter, $\hbar _f$, $\hbar _g$, $\hbar _h$, $\hbar _k$, $\hbar _\theta$ and $\hbar _\Psi$ are the nonzero auxiliary parameter and $\textit{N}_f$, $\textit{N}_g$, $\textit{N}_h$, $\textit{N}_k$, $\textit{N}_\theta$ and $\textit{N}_\Psi$ are the nonlinear parameters. For $\beta = 0$ and 1, we have

49 $$\begin{aligned} \begin{aligned}{}&{\bar{f}}({\eta} ,0) = f_o({\eta} ), \,\,{\bar{f}}({\eta} ,1) = f({\eta} ), \\&{\bar{g}}({\eta} ,0) = g_o({\eta} ), \,\,{\bar{g}}({\eta} ,1) = g({\eta} ), \\&{\bar{h}}({\eta} ,0) = h_o({\eta} ),\,\, {\bar{h}}({\eta} ,1) = h({\eta} ), \\&{\bar{k}}({\eta} ,0) = k_o({\eta} ), \,\,{\bar{k}}({\eta} ,1) = k({\eta} ), \\&{\bar{\theta }}({\eta} ,0) = \theta _o({\eta} ), \,\,{\bar{\theta }}({\eta} ,1) = \theta ({\eta} ), \\&{\bar{\Psi }}({\eta} ,0) = \Psi _o({\eta} ), {\bar{\Psi }}({\eta} ,1) = \Psi ({\eta} ), \end{aligned} \end{aligned}$$

so we can say that as $\beta$ varies from 0 to 1, ${\bar{f}}({\eta} ,0)$, ${\bar{g}}({\eta} ,0)$, ${\bar{h}}({\eta} ,0)$, ${\bar{k}}({\eta} ,0)$, ${\bar{\theta }}({\eta} ,0)$, ${\bar{\Psi }}({\eta} ,0)$ varies from initial guesses $f_0({\eta} )$, $g_0({\eta} )$, $h_0({\eta} )$, $k_0({\eta} )$, $\theta _0({\eta} )$ and $\Psi _0({\eta} )$ to exact solution $f({\eta} )$, $g({\eta} )$, $h({\eta} )$, $k({\eta} )$, $\theta ({\eta} )$ and $\Psi ({\eta} )$ respectively.

Taylor’s series expansion of these functions yields:

50 $$\begin{aligned} f({\eta} ;\beta )= \,\,& {} f_0({\eta} ) + \sum _{m=1}^{\infty }\beta ^mf_m({\eta} ), \end{aligned}$$

51 $$\begin{aligned} g({\eta} ;\beta )= \,\,& {} g_0({\eta} ) + \sum _{m=1}^{\infty }\beta ^mg_m({\eta} ), \end{aligned}$$

52 $$\begin{aligned} h({\eta} ;\beta )= \,\,& {} h_0({\eta} ) + \sum _{m=1}^{\infty }\beta ^mh_m({\eta} ), \end{aligned}$$

53 $$\begin{aligned} k({\eta} ;\beta )= \,\,& {} k_0({\eta} ) + \sum _{m=1}^{\infty }\beta ^mk_m({\eta} ), \end{aligned}$$

54 $$\begin{aligned} \theta ({\eta} ;\beta )= \,\,& {} \theta _0({\eta} ) + \sum _{m=1}^{\infty }\beta ^m\theta _m({\eta} ), \end{aligned}$$

55 $$\begin{aligned} \Psi ({\eta} ;\beta )= \,\,& {} \Psi _0({\eta} ) + \sum _{m=1}^{\infty }\beta ^m\Psi _m({\eta} ), \end{aligned}$$

56 $$\begin{aligned} f_m({\eta} )= \,\,& {} \frac{1}{m!}\frac{\partial ^{m}f({\eta} ;\beta )}{\partial \eta ^{m}}\bigg |_{\beta = 0}, g_m({\eta} ) = \frac{1}{m!}\frac{\partial ^{m}g({\eta} ;\beta )}{\partial \eta ^{m}}\bigg |_{\beta = 0},h_m({\eta} ) = \frac{1}{m!}\frac{\partial ^{m}m({\eta} ;\beta )}{\partial \eta ^{m}}\bigg |_{\beta = 0}, \nonumber \\ k_m({\eta} )= \,\,& {} \frac{1}{m!}\frac{\partial ^{m}n({\eta} ;\beta )}{\partial \eta ^{m}}\bigg |_{\beta = 0}, \theta _m({\eta} ) = \frac{1}{m!}\frac{\partial ^{m}\theta ({\eta} ;\beta )}{\partial \eta ^{m}}\bigg |_{\beta = 0}, \Psi _m({\eta} ) = \frac{1}{m!}\frac{\partial ^{m}\Psi ({\eta} ;\beta )}{\partial \eta ^{m}}\bigg |_{\beta = 0}, \end{aligned}$$

it should be noted that the convergence of above series strongly depends upon $\hbar _f$, $\hbar _g$, $\hbar _h$, $\hbar _k$, $\hbar _\theta$ and $\hbar _\Psi$.

Assuming that these nonzero auxiliary parameters are chosen so that Eqs. (35–40) converges at $\beta = 1$. Therefore one can obtain

57 $$\begin{aligned} f({\eta} )= \,\,& {} f_0({\eta} ) + \sum _{m=1}^{\infty }f_m({\eta} ), \end{aligned}$$

58 $$\begin{aligned} g({\eta} )= \,\,& {} g_0({\eta} ) + \sum _{m=1}^{\infty }g_m({\eta} ), \end{aligned}$$

59 $$\begin{aligned} h({\eta} )= \,\,& {} h_0({\eta} ) + \sum _{m=1}^{\infty }h_m({\eta} ), \end{aligned}$$

60 $$\begin{aligned} k({\eta} )= \,\,& {} k_0({\eta} ) + \sum _{m=1}^{\infty }k_m({\eta} ), \end{aligned}$$

61 $$\begin{aligned} \theta ({\eta} )= \,\,& {} \theta _0({\eta} ) + \sum _{m=1}^{\infty }\theta _m({\eta} ), \end{aligned}$$

62 $$\begin{aligned} \Psi ({\eta} )= \,\,& {} \Psi _0({\eta} ) + \sum _{m=1}^{\infty }\Psi _m({\eta} ), \end{aligned}$$

Differentiating the deformation Eqs. (35–40) $m-times$ with respect to $\beta$ and putting $\beta = 0$, we have

63 $$\begin{aligned} \ell _f[f_m({\eta} ) - \chi _mf_{m-1}({\eta} )]= \,\,& {} \hbar _f R_{f,m}({\eta} ), \end{aligned}$$

64 $$\begin{aligned} \ell _g[g_m({\eta} ) - \chi _mg_{m-1}({\eta} )]= \,\,& {} \hbar _g R_{g,m}({\eta} ), \end{aligned}$$

65 $$\begin{aligned} \ell _h[h_m({\eta} ) - \chi _mh_{m-1}({\eta} )]= \,\,& {} \hbar _h R_{h,m}({\eta} ), \end{aligned}$$

66 $$\begin{aligned} \ell _k[k_m({\eta} ) - \chi _mk_{m-1}({\eta} )]= \,\,& {} \hbar _k R_{k,m}({\eta} ), \end{aligned}$$

67 $$\begin{aligned} \ell _\theta [\theta _m({\eta} ) - \chi _m\theta _{m-1}({\eta} )]= \,\,& {} \hbar _\theta R_{\theta ,m}({\eta} ), \end{aligned}$$

68 $$\begin{aligned} \ell _\Psi [\Psi _m({\eta} ) - \chi _m\Psi _{m-1}({\eta} )]= \,\,& {} \hbar _\Psi R_{\Psi ,m}({\eta} ), \end{aligned}$$

subject to the boundary conditions

69 $$\begin{aligned} \begin{aligned} f_m(0)&= 0, \,\,f'_m(0) = 0,\,\, g_m(0) = 0,\,\, h_m(0) = 0,\,\, k_m(0) = 0, \,\,\theta _m(0) = 0, \,\,\Psi _m(0) = 1, \\ f_m(1)&= 0.5, \,\,f'_m(1) = 0,\,\,g_m(1) = S, \,\,h_m(1) = 1,\,\, k_m(1) = 1, \,\,\theta _m(1) = 0,\,\, \Psi _m(1) = 0, \end{aligned} \end{aligned}$$

where

70 $$\begin{aligned} R_{f,m}({\eta} )= \,\,& {} \aleph ^2_{b}f''''_{m-1}({\eta} ) - \aleph ^3_{b}\bigg [3f''_{m-1}({\eta} ) + \eta f'''_{m-1}({\eta} ) - 2\sum _{j=0}^{m-1}f_{j}({\eta} ) f'''_{m-j-1} \nonumber \\&+\,\, 2\xi ^{2}_{z}\xi _{mag}\sum _{j=0}^{m-1}m_{j}({\eta} )\bigg ({\eta} h''_{m-j-1} + h'_{m-j-1} + 2h_{m-j-1}f''_{m-j-1} - 2f_{m-j-1}h''_{m-j-1}\bigg ) \bigg ] \nonumber \\&+\,\, 2\aleph ^2_{a}\bigg (\sum _{j=0}^{m-1}g_{j}({\eta} ) g'_{m-j-1}({\eta} ) - \xi ^{2}_{\theta }\sum _{j=0}^{m-1}n_{j}({\eta} )k'_{m-j-1}({\eta} )\bigg ), \end{aligned}$$

71 $$\begin{aligned} R_{g,m}({\eta} )= \,\,& {} g''_{m-1}({\eta} ) - \xi _{sq}\bigg [\eta g'_{m-1}({\eta} ) + 2g_{m-1} + 2\sum _{j=0}^{m-1}g_{j}({\eta} ) f'_{m-j-1}({\eta} ) - 2\sum _{j=0}^{m-1}f_{j}({\eta} ) g'_{m-j-1}({\eta} ) \nonumber \\&+\,\, 2\xi _{z}\xi _{\theta }\bigg ( \sum _{j=0}^{m-1}h_{j}({\eta} ) k'_{m-j-1}({\eta} ) - \sum _{j=0}^{m-1}k_{j}({\eta} )h'_{m-j-1}({\eta} )\bigg )\bigg ], \end{aligned}$$

72 $$\begin{aligned} R_{h,m}({\eta} )= \,\,& {} h''_{m-1}({\eta} ) - \xi _{mag}\bigg [\eta h'_{m-1}({\eta} ) + h_{m-1} - 2\sum _{j=0}^{m-1}f_{j}({\eta} )h'_{m-j-1}({\eta} ) + 2\sum _{j=0}^{m-1}h_{j}({\eta} ) f'_{m-j-1}({\eta} ) \nonumber \\&-\,\, \sum _{j=0}^{m-1}k_{j}({\eta} )h'_{m-j-1}({\eta} )\bigg )\bigg ], \end{aligned}$$

73 $$\begin{aligned} R_{k,m}({\eta} )= \,\,& {} \xi _{\theta } k''_{m-1}({\eta} ) - \xi _{mag}\xi _{\theta }\bigg [\eta h'_{m-1}({\eta} ) + 2h_{m-1} - 2\sum _{j=0}^{m-1}f_{j}({\eta} )h'_{m-j-1}({\eta} )\bigg ] \nonumber \\&-\,\, 2\xi _{z}\sum _{j=0}^{m-1}h_{j}({\eta} ) g'_{m-j-1}({\eta} ), \end{aligned}$$

74 $$\begin{aligned} R_{\theta ,m}({\eta} )= \,\,& {} \theta ''_{m-1}({\eta} ) + \xi _{sq}\xi _{pr}\bigg (2\sum _{j=0}^{m-1}f_{j}({\eta} )\theta '_{m-j-1}({\eta} ) - \eta \theta '_{j}({\eta} )\bigg ) - \xi _{ec}\xi _{hm}\xi _{pm}\bigg ({\eta} h'_{m-1}({\eta} ) \nonumber \\&+\,\, h_{m-1}({\eta} ) - 2\sum _{j=0}^{m-1}f_{j}({\eta} )h'_{m-j-1}({\eta} ) \bigg ), \end{aligned}$$

75 $$\begin{aligned} R_{\Psi ,m}({\eta} )= \,\,& {} \Psi ''_{m-1}({\eta} ) + \xi _{sq}\xi _{pr}\bigg (2\sum _{j=0}^{m-1}f_{j}({\eta} )\Psi '_{m-j-1}({\eta} ) - \eta \Psi '_{j}({\eta} )\bigg ) - \xi _{ec}\xi _{hm}\xi _{sm}\bigg ({\eta} h'_{m-1}({\eta} ) \nonumber \\&+\,\, h_{m-1}({\eta} ) - 2\sum _{j=0}^{m-1}f_{j}({\eta} )h'_{m-j-1}({\eta} ) \bigg ), \end{aligned}$$

and $$\chi _m = \bigg \{1,\,\, if\,\, m > 1 ,\,\,\,\,\, and\,\, 0,\,\, m = 1$$.

Finally, the general solution of Eqs. (59–64) can be written as

76 $$\begin{aligned} f_m({\eta} )= \,\,& {} \int _{0}^{\eta }\int _{0}^{\eta }\int _{0}^{\eta }\int _{0}^{\eta }\hbar _f R_{f,m}(z)dzdzdzdzdz + \chi _mf_{m-1} + \zeta _{1}\eta ^3 + \zeta _{2}\eta ^2 + \zeta _{3}\eta + \zeta _{4}, \end{aligned}$$

77 $$\begin{aligned} g_m({\eta} )= \,\,& {} \int _{0}^{\eta }\int _{0}^{\eta }\hbar _g R_{g,m}(z)dzdz + \chi _mg_{m-1} + \zeta _{5}\eta + \zeta _{6}, \end{aligned}$$

78 $$\begin{aligned} h_m({\eta} )= \,\,& {} \int _{0}^{\eta }\int _{0}^{\eta }\hbar _h R_{h,m}(z)dzdz + \chi _mh_{m-1} + \zeta _{7}\eta + \zeta _{8}, \end{aligned}$$

79 $$\begin{aligned} k_m({\eta} )= \,\,& {} \int _{0}^{\eta }\int _{0}^{\eta }\hbar _k R_{k,m}(z)dzdz + \chi _mk_{m-1} + \zeta _{9}\eta + \zeta _{10}, \end{aligned}$$

80 $$\begin{aligned} \theta _m({\eta} )= \,\,& {} \int _{0}^{\eta }\int _{0}^{\eta }\hbar _\theta R_{\theta ,m}(z)dzdz + \chi _m\theta _{m-1} + \zeta _{11}\eta + \zeta _{12}, \end{aligned}$$

81 $$\begin{aligned} \Psi _m({\eta} )= \,\,& {} \int _{0}^{\eta }\int _{0}^{\eta }\hbar _\Psi R_{\Psi ,m}(z)dzdz + \chi _m\Psi _{m-1} + \zeta _{13}\eta + \zeta _{14}, \end{aligned}$$

and so the exact solution $f({\eta} )$, $g({\eta} )$, $h({\eta} )$, $k({\eta} )$, $\theta ({\eta} )$ and $\Psi ({\eta} )$ becomes

82 $$\begin{aligned} \begin{aligned}{}&f({\eta} ) \approx \sum _{n=0}^{m}f_n({\eta} ), \,\,g({\eta} ) \approx \sum _{n=0}^{m}g_n({\eta} ),\,\, h({\eta} ) \approx \sum _{n=0}^{m}h_n({\eta} ), \\&k({\eta} ) \approx \sum _{n=0}^{m}k_n({\eta} ),\,\, \theta ({\eta} ) \approx \sum _{n=0}^{m}\theta _n({\eta} ) ,\,\, \Psi ({\eta} ) \approx \sum _{n=0}^{m}\Psi _n({\eta} ). \end{aligned} \end{aligned}$$

### Optimal convergence control parameters

It must be remarked that the series solutions (59–64) contain the nonzero auxiliary parameters $\hbar _f$, $\hbar _g$,, $\hbar _h$, $\hbar _k$, $\hbar _\theta$ and $\hbar _\Psi$ which determine the convergence region and also rate of the homotopy series solutions. To obtain the optimal values of $\hbar _f$, $\hbar _g$, $\hbar _h$, $\hbar _k$, $\hbar _\theta$ and $\hbar _\Psi$ here the so called average residual error defined by Liao^16^ were used as:

83 $$\begin{aligned} \varepsilon ^{f}_{m}= \,\,& {} \frac{1}{\zeta + 1}\sum _{j=0}^{\zeta }\bigg [\textit{N}_f\bigg (\sum _{i=0}^{m}{\bar{f}}({\eta} ), \sum _{i=0}^{m}{\bar{g}}({\eta} ), \sum _{i=0}^{m}{\bar{h}}({\eta} ), \sum _{i=0}^{m}{\bar{k}}({\eta} )\bigg )_{n=j\delta n}\bigg ]^2d\eta , \end{aligned}$$

84 $$\begin{aligned} \varepsilon ^{g}_{m}= \,\,& {} \frac{1}{\zeta + 1}\sum _{j=0}^{\zeta }\bigg [\textit{N}_g\bigg (\sum _{i=0}^{m}{\bar{f}}({\eta} ), \sum _{i=0}^{m}{\bar{g}}({\eta} ), \sum _{i=0}^{m}{\bar{h}}({\eta} ), \sum _{i=0}^{m}{\bar{k}}({\eta} )\bigg )_{n=j\delta n}\bigg ]^2d\eta , \end{aligned}$$

85 $$\begin{aligned} \varepsilon ^{h}_{m}= \,\,& {} \frac{1}{\zeta + 1}\sum _{j=0}^{\zeta }\bigg [\textit{N}_m\bigg (\sum _{i=0}^{m}{\bar{f}}({\eta} ), \sum _{i=0}^{m}{\bar{h}}({\eta} ), \sum _{i=0}^{m}{\bar{k}}({\eta} )\bigg )_{n=j\delta n}\bigg ]^2d\eta , \end{aligned}$$

86 $$\begin{aligned} \varepsilon ^{k}_{m}= \,\,& {} \frac{1}{\zeta + 1}\sum _{j=0}^{\zeta }\bigg [\textit{N}_n\bigg (\sum _{i=0}^{m}{\bar{f}}({\eta} ), \sum _{i=0}^{m}{\bar{h}}({\eta} ), \sum _{i=0}^{m}{\bar{k}}({\eta} )\bigg )_{n=j\delta n}\bigg ]^2d\eta , \end{aligned}$$

87 $$\begin{aligned} \varepsilon ^{\theta }_{m}= \,\,& {} \frac{1}{\zeta + 1}\sum _{j=0}^{\zeta }\bigg [\textit{N}_\theta \bigg (\sum _{i=0}^{m}{\bar{f}}({\eta} ), \sum _{i=0}^{m}{\bar{\theta }}({\eta} ),\sum _{i=0}^{m}{\bar{\Psi }}({\eta} )\bigg )_{n=j\delta n}\bigg ]^2d\eta , \end{aligned}$$

88 $$\begin{aligned} \varepsilon ^{\Psi }_{m}= \,\,& {} \frac{1}{\zeta + 1}\sum _{j=0}^{\zeta }\bigg [\textit{N}_\Psi \bigg (\sum _{i=0}^{m}{\bar{f}}({\eta} ), \sum _{i=0}^{m}{\bar{\theta }}({\eta} ),\sum _{i=0}^{m}{\bar{\Psi }}({\eta} )\bigg )_{n=j\delta n}\bigg ]^2d\eta , \end{aligned}$$

Due to Liao^16^

89 $$\begin{aligned} \varepsilon ^{t}_{m} = \varepsilon ^{f}_{m} + \varepsilon ^{g}_{m} + \varepsilon ^{h}_{m} + \varepsilon ^{k}_{m} + \varepsilon ^{\theta }_{m} + \varepsilon ^{\Psi }_{m}, \end{aligned}$$

where $\varepsilon ^{t}_{m}$ is the total squared residual error. Total average squared residual error is minimized by employing Mathematica package **BVPh 2.0**^16^.

## Physical quantities of interest

### Torque exerted by fluid on the plates

The frictional moment or torque which the fluid exerts on the upper plate is given^26^

$$\begin{aligned} T_{upper} = 2\pi \mu \int _{0}^{c}r^{2}\bigg (\frac{\partial v}{\partial z}\bigg )_{z=D(t)}dr, \end{aligned}$$

but $v = \frac{\Omega _{u} r}{1 - \alpha t}g({\eta} )$ so the above equation becomes

$$\begin{aligned}T_{upper} = \frac{\pi \mu \Omega _{u} c^{4}}{2l(1-\alpha t)^{\frac{3}{2}}}g'(1)\end{aligned}$$,

90 $$\begin{aligned} \text {or}\,\,\, T^{*}_{upper} = g'(1)\,\,\,\,\,\,\,\, \text {where}\,\,\,\, T^{*}_{upper} = \frac{2l(1-\alpha t)^{\frac{3}{2}}}{\pi \mu \Omega _{u} c^4}. \end{aligned}$$

For the lower plate the corresponding result is

91 $$\begin{aligned} T^{*}_{lower} = g'(0), \end{aligned}$$

### The pressure or normal force of the fluid on plates

According to HAM za et al.^26^, the pressure or the normal force which the fluid exerts on the upper plate is given as:

92 $$\begin{aligned} F = 2\pi \bigg [ \int _{0}^{a}rP(r,1,t)dr - \int _{0}^{a}rP^{+}(r,1,t)dr\bigg ], \end{aligned}$$

where $P^+(r,1,t)$ in for conditions on the side of the plate and *p*(*r*, 1, *t*) denotes the pressure at the edge of the disc at time *t*^26^. Let us assume that $\frac{\partial P^+(r,1,t)}{\partial r} = 0$ and using Eq. (8), we get

$$\begin{aligned} \begin{aligned} \frac{\partial p}{\partial r} = \frac{\rho r}{(1-\alpha t)^2}\bigg [ \frac{\alpha \nu }{2l^2}\xi _{vis} f''' + \Omega _{u}^2g^2 - \frac{\alpha ^2}{4}&\bigg ( 2f' + \eta f'' + f'' - 2ff'' \bigg ) \\&- \frac{2}{\rho \mu _2}\bigg ( \frac{\alpha ^2M^{2}_{o}}{4l^2}hh'' + N^{2}_{o}k^2 \bigg )\bigg ], \end{aligned} \end{aligned}$$

93 $$\begin{aligned} \begin{aligned} \Upsilon (t,\eta )= \frac{1}{(1-\alpha t)^2}\bigg [ \frac{\alpha \nu }{2l^2}\xi _{vis} f''' + \Omega _{u}^2g^2 - \frac{\alpha ^2}{4}&\bigg ( 2f' + \eta f'' + f'' - 2ff'' \bigg ) \\&- \frac{2}{\rho \mu _2}\bigg ( \frac{\alpha ^2M^{2}_{o}}{4l^2}hh'' + N^{2}_{o}k^2 \bigg )\bigg ], \end{aligned} \end{aligned}$$

where $\Upsilon (t,\eta ) = \frac{1}{\rho r}\frac{\partial p}{\partial r}$. using Eqs. (25), (26) , we have

$$\begin{aligned} F = \frac{\pi \rho \alpha ^2 a^4}{16(1-\alpha t)^2}\bigg [ 2\xi ^{2}_{z}h''(1) - \aleph ^{-1}_{b}\xi _{vis} f'''(1) - \bigg (\frac{\xi _{rad}}{\xi _{sq}}\bigg )^2\bigg (S^2 - 2\xi ^{2}_{\theta }\bigg )\bigg ], \end{aligned}$$

94 $$\begin{aligned} F_{pres} = \bigg [ 2\xi ^{2}_{z}h''(1) - \aleph ^{-1}_{b}\xi _{vis} f'''(1) - \bigg (\frac{\xi _{rad}}{\xi _{sq}}\bigg )^2\bigg (S^2 - 2\xi ^{2}_{\theta }\bigg )\bigg ], \end{aligned}$$

where $F_{pres} = \frac{16(1-\alpha t)^2}{\pi \rho \alpha ^2 a^4}$, Which is the dimensionless pressure on the upper plate. The positive or negative numerical values of $F_{pres}$ will be according the force acting by the fluid on the upper plate is in the positive or negative direction of the z-axis respectively.

## Error analysis

**Figure 1:**
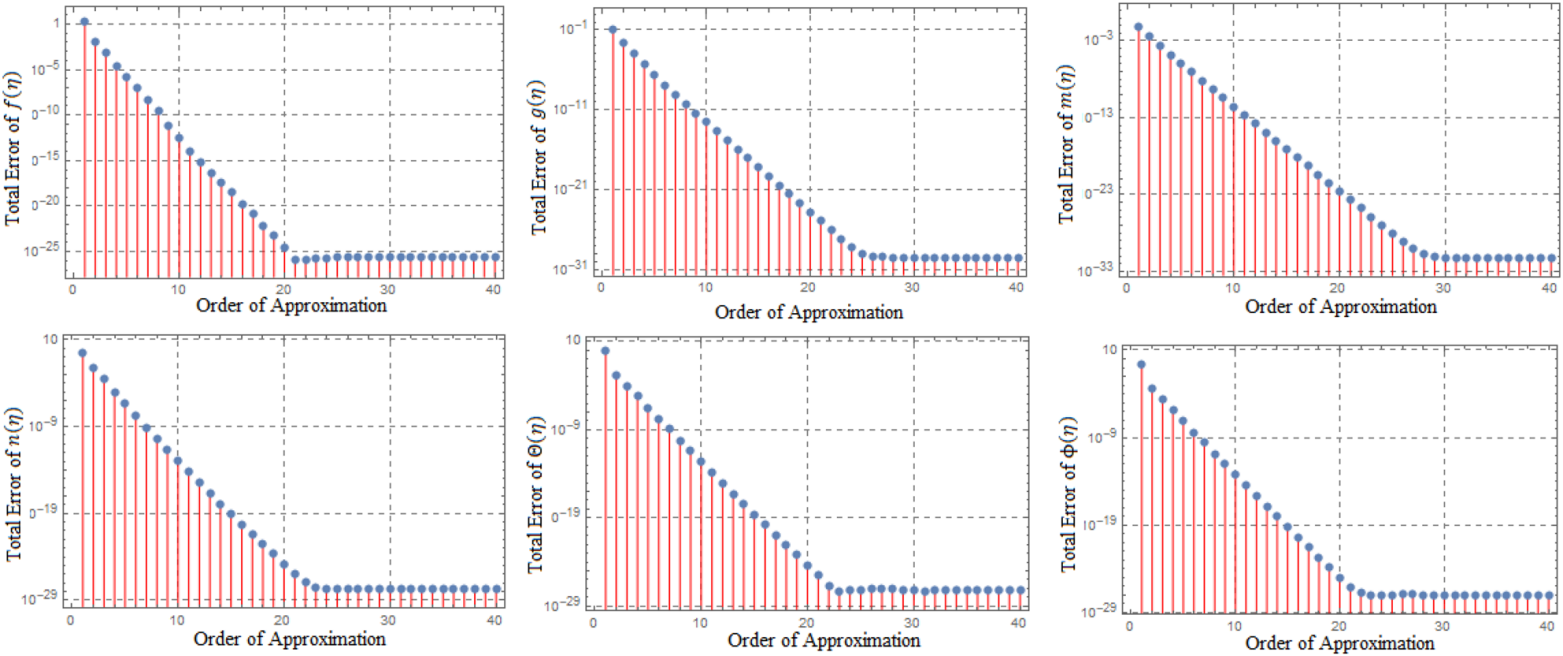
Error profile of $f({\eta} )$, $g({\eta} )$, $m({\eta} )$, $n({\eta} )$, $\theta ({\eta} )$ and $\phi ({\eta} )$ for fixed values of $\xi _{a} = 0.1$, $\xi _{s} = 0.1$, $\xi _{z} = 0.2$, $\xi _{\theta } = 0.5$, $\xi _{em} = 0.1$, $\xi _{pr} = 3$, $\xi _{hm} = 0.1$, $\xi _{pm} = 1.5$, $\xi _{sm} = 1$, $\xi _{ec} = 0.2$, $\xi _{v} = 0.5$ and $S = 1$.

**Figure 2:**
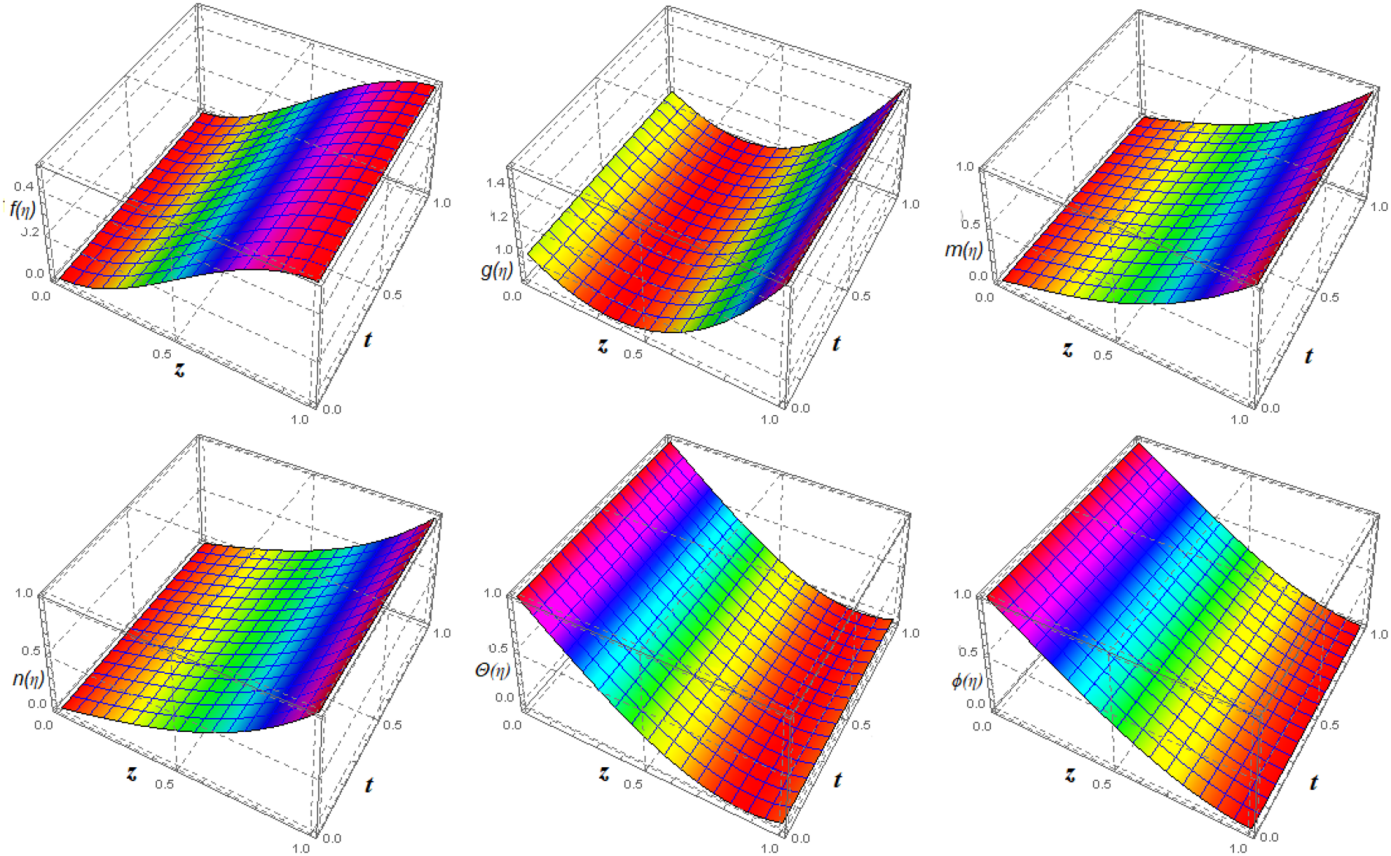
Error profile of $f({\eta} )$, $g({\eta} )$, $m({\eta} )$, $n({\eta} )$, $\theta ({\eta} )$ and $\phi ({\eta} )$ for fixed values of $\xi _{a} = 0.1$, $\xi _{s} = 0.1$, $\xi _{z} = 0.2$, $\xi _{\theta } = 0.5$, $\xi _{em} = 0.1$, $\xi _{pr} = 3$, $\xi _{hm} = 0.1$, $\xi _{pm} = 1.5$, $\xi _{sm} = 1$, $\xi _{ec} = 0.2$, $\xi _{v} = 0.5$ and $S = 1$.

Error assessments are performed to ensure that the derived system of ODEs is analytically accurate with the lowest residual errors. The validity of HAM techniques utilising the highest residual error $10^{-38}$ is also investigated in this study. Approximations of the $30{th}$ order of approximation are used in the study. Error analyses in Fig. 1 and tabulated data in Tables 1, 2, 3, 4, 5, 6, 7, 8, 9, 10, 11 and 12 are offered to validate the legitimacy of the findings for the various included physical parameters. Figure 2 is drawn to show the 3*D*-view of the velocity field components, magnetic field components and heat/mass distribution.

**Table 1:** Optimal values of convergence control parameters versus different orders of approximation with fixed values of $\xi _{rad} = 0.1$, $\xi _{sq} = 0.1$, $\xi _{z} = 0.2$, $\xi _{\theta } = 0.5$, $\xi _{mag} = 0.1$, $\xi _{pr} = 3$, $\xi _{hm} = 0.1$, $\xi _{pm} = 1.5$, $\xi _{sm} = 1$, $\xi _{ec} = 0.2$, $\xi _{vis} = 0.5$ and $S = 1$.

Order	$h_{f}$	$h_{g}$	$h_{h}$	$h_{k}$	$h_{\theta }$	$h_{\Psi }$	$\varepsilon ^{t}_{m}$
2	$-\,182.02$	$-\,2.8096$	$-\,1.8780$	$-\,1.8760$	$-\,1.8468$	$-\,1.8428$	$2.0114\times 10^{-7}$
3	$-\,190.87$	$-\,1.9148$	$-\,0.9691$	$-\,0.9812$	$-\,0.9728$	$-\,0.9712$	$1.1235\times 10^{-10}$
4	$-\,191.92$	$-1.8999$	$-\,0.9952$	$-\,1.0173$	$-\,0.9665$	$-\,0.9665$	$4.3529\times 10^{-13}$
5	$-\,190.97$	$-1.9025$	$-\,0.9882$	$-\,0.9810$	$-\,0.9530$	$-\,0.9498$	$-\,2.5293\times 10^{-15}$
6	$-191.79$	$-1.9184$	$-\,0.9828$	$-\,0.9456$	$-\,0.9498$	$-\,0.9412$	$-\,6.6548\times 10^{-17}$

**Table 2:** Total residual error for different order of approximations taking fixed values of $\xi _{rad} = 0.1$, $\xi _{sq} = 0.1$, $\xi _{z} = 0.2$, $\xi _{\theta } = 0.5$, $\xi _{mag} = 0.3$, $\xi _{pr} = 0.2$, $\xi _{hm} = 1$, $\xi _{pm} = 0.2$, $\xi _{sm} = 1$, $\xi _{ec} = 0.1$, $\xi _{vis} = 0.3$ and $S = 0.5$.

*m*	${\epsilon ^{f}}_{m}$	${\epsilon ^{g}}_{m}$	${\epsilon ^{h}}_{m}$	${\epsilon ^{k}}_{m}$	${\epsilon ^{\theta }}_{m}$	${\epsilon ^{\Psi }}_{m}$
2	$1.231\times 10^{-7}$	$2.864\times 10^{-6}$	$2.245\times 10^{-6}$	$2.752\times 10^{-5}$	$8.534\times 10^{-9}$	$5.234\times 10^{-6}$
5	$3.342\times 10^{-14}$	$4.127\times 10^{-13}$	$3.421\times 10^{-14}$	$2.531\times 10^{-13}$	$3.274\times 10^{-17}$	$3.819\times 10^{-15}$
8	$4.173\times 10^{-21}$	$3.223\times 10^{-20}$	$3.425\times 10^{-21}$	$4.426\times 10^{-20}$	$4.342\times 10^{-24}$	$3.124\times 10^{-21}$
11	$7.421\times 10^{-28}$	$8.658\times 10^{-27}$	$1.238\times 10^{-27}$	$3.153\times 10^{-27}$	$3.446\times 10^{-31}$	$2.456\times 10^{-29}$c
14	$2.642\times 10^{-34}$	$2.451\times 10^{-32}$	$1.395\times 10^{-32}$	$3.327\times 10^{-32}$	$2.148\times 10^{-35}$	$3.469\times 10^{-33}$
17	$2.351\times 10^{-34}$	$1.478\times 10^{-32}$	$1.868\times 10^{-32}$	$2.231\times 10^{-32}$	$2.424\times 10^{-35}$	$4.324\times 10^{-33}$
20	$2.351\times 10^{-34}$	$1.478\times 10^{-32}$	$8.223\times 10^{-33}$	$2.925\times 10^{-32}$	$1.941\times 10^{-35}$	$5.970\times 10^{-33}$
23	$2.351\times 10^{-34}$	$1.478\times 10^{-32}$	$1.068\times 10^{-32}$	$2.741\times 10^{-32}$	$1.760\times 10^{-35}$	$5.691\times 10^{-33}$
26	$2.351\times 10^{-34}$	$1.478\times 10^{-32}$	$1.377\times 10^{-32}$	$3.049\times 10^{-32}$	$1.700\times 10^{-35}$	$5.845\times 10^{-33}$
30	$2.351\times 10^{-34}$	$1.478\times 10^{-32}$	$1.038\times 10^{-32}$	$2.432\times 10^{-32}$	$1.941\times 10^{-35}$	$6.654\times 10^{-33}$

**Table 3:** Computations for $f({\eta })$ ,$g({\eta })$, $h({\eta })$, $k({\eta })$, $\theta ({\eta })$, $\Psi ({\eta })$ with $\xi _{rad} = 0.1$, $\xi _{sq} = 0.1$, $\xi _{z} = 0.2$, $\xi _{\theta } = 0.5$, $\xi _{mag} = 0.3$, $\xi _{pr} = 0.2$, $\xi _{hm} = 1$, $\xi _{pm} = 0.2$, $\xi _{sm} = 1$, $\xi _{ec} = 0.1$, $\xi _{vis} = 0.3$, $S = 0.5$ and various values of $\eta$.

$\eta$	HAM result					BVP4c result				
	$f({\eta} )$	$g({\eta} )$	$h({\eta} )$	$\theta ({\eta} )$	$\Psi ({\eta} )$	$f({\eta} )$	$g({\eta} )$	$h({\eta} )$	$\theta ({\eta} )$	$\Psi ({\eta} )$
0	0	1	0	1	1	0	1	0	1	1
0.1001	0.0261	0.9267	0.0899	0.8987	0.8974	0.0261	0.9264	0.0899	0.8989	0.8977
0.2002	0.0668	0.8497	0.1911	0.7987	0.7978	0.0669	0.8499	0.1912	0.7989	0.7978
0.3003	0.1258	0.7703	0.2716	0.6989	0.6954	0.1259	0.7704	0.2717	0.6989	0.6955
0.4004	0.1839	0.7100	0.3654	0.5986	0.5944	0.1839	0.7101	0.3655	0.5987	0.5945
0.5005	0.2582	0.6579	0.4619	0.4983	0.4938	0.2582	0.6579	0.4619	0.4983	0.4938
0.6006	0.3324	0.6245	0.5723	0.3994	0.3946	0.3325	0.6246	0.5724	0.3995	0.3947
0.7007	0.3985	0.5875	0.6765	0.2993	0.2951	0.3986	0.5876	0.6766	0.2994	0.2952
0.8008	0.4622	0.5568	0.7845	0.1994	0.1959	0.4623	0.5569	0.7846	0.1995	0.1959
0.9009	0.4981	0.5315	0.8965	0.0997	0.0976	0.4982	0.5316	0.8966	0.0998	0.0977
1	0.5	0.5	1	0	0	0.5	0.5	1	0	0

**Table 4:** Convergence of HAM solution for different orders of approximation for $f''(0)$, $-g'(0)$, $-h'(0)$, $-n'(0)$, $-\theta ' (0)$ and $-\Psi ' (0)$ when $\xi _{rad} = 0.1$, $\xi _{sq} = 0.1$, $\xi _{z} = 0.2$, $\xi _{\theta } = 0.5$, $\xi _{mag} = 0.3$, $\xi _{pr} = 0.2$, $\xi _{hm} = 1$, $\xi _{pm} = 0.2$, $\xi _{sm} = 1$, $\xi _{ec} = 0.1$, $\xi _{vis} = 0.3$ and $S = 0.5$.

*m*	$f''(0)$	$-\,g'(0)$	$-\,h'(0)$	$-\,k'(0)$	$-\,\theta ' (0)$	$-\,\Psi ' (0)$
1	3.30248492	0.76935826	$-0.76249352$	$-0.86198432$	1.01629436	1.00261843
6	3.30248445	0.76935839	$-0.76249348$	$-0.861984256$	1.01629429	1.00261862
12	3.30248472	0.76935856	$-0.76249352$	$-0.861984261$	1.01629435	1.00261869
18	3.30248472	0.76935856	$-0.76249352$	$-0.861984261$	1.01629435	1.00261869
24	3.30248472	0.76935856	$-0.76249352$	$-0.861984261$	1.01629435	1.00261869
30	3.30248472	0.76935856	$-0.76249352$	$-0.861984261$	1.01629435	1.00261869

**Table 5:** Fluid pressure and torques on upper plate with $\xi _{rad} = 0.1$, $\xi _{z} = 0.2$, $\xi _{\theta } = 0.5$, $\xi _{mag} = 0.1$, $\xi _{pr} = 3$, $\xi _{hm} = 1$, $\xi _{pm} = 0.2$, $\xi _{sm} = 1$, $\xi _{ec} = 2$, $\xi _{vis} = 0.3$, $S = 1$ and various values of $\xi _{sq}$.

$\xi _{sq}$	HAM result		BVP4c result		%Error = $\vert \frac{N.R - H.R}{N.R}\vert$	
	$F_{pres}$	$g'(1)$	$F_{pres}$	$g'(1)$	$F_{pres}$	$g'(1)$
0.1	19.2226	$-\,0.4558$	19.2225	$-\,0.4562$	$5.20224\times 10^{-6}$	$2.19346\times 10^{-3}$
0.5	5.2753	$-\,1.7021$	5.2752	$-\,0.4724$	$1.89566\times 10^{-4}$	$5.87475\times 10^{-4}$
1	3.4494	$-\,2.6614$	3.4493	$-\,0.5128$	$2.89914\times 10^{-4}$	$3.75728\times 10^{-4}$
1.5	2.8340	$-\,3.3410$	2.8339	$-\,0.3400$	$3.52871\times 10^{-4}$	$2.99303\times 10^{-4}$

**Table 6:** Fluid pressure and torques on upper plate with $\xi _{rad} = 0.1$, $\xi _{sq} = 1.5$, $\xi _{z} = 0.2$, $\xi _{\theta } = 0.5$, $\xi _{mag} = 0.1$, $\xi _{pr} = 3$, $\xi _{hm} = 1$, $\xi _{pm} = 0.2$, $\xi _{sm} = 1$, $\xi _{ec} = 2$, $S = 1$ and various values of $\xi _{vis}$.

$\xi _{vis}$	HAM result		BVP4c result		%Error = $\vert \frac{N.R - H.R}{N.R}\vert$	
	$F_{pres}$	$g'(1)$	$F_{pres}$	$g'(1)$	$F_{pres}$	$g'(1)$
0.1	1.9722	$-\,5.5855$	1.9721	$-\,5.5856$	$5.07074\times 10^{-4}$	$1.79032\times 10^{-4}$
0.5	3.6515	$-\,2.4989$	3.6514	$-\,2.4990$	$2.73868\times 10^{-4}$	$4.00160\times 10^{-4}$
1	5.6661	$-\,1.5789$	5.6660	$-\,1.5790$	$1.76491\times 10^{-4}$	$6.33312\times 10^{-4}$
1.5	7.6712	$-\,1.1632$	7.6711	$-\,1.1633$	$1.30359\times 10^{-4}$	$8.59623\times 10^{-4}$

**Table 7:** Fluid pressure and torques on upper plate with $\xi _{sq} = 0.1$, $\xi _{z} = 0.2$, $\xi _{\theta } = 0.5$, $\xi _{mag} = 0.3$, $\xi _{pr} = 3$, $\xi _{hm} = 1$, $\xi _{pm} = 0.2$, $\xi _{sm} = 1$, $\xi _{ec} = 2$, $\xi _{vis} = 0.3$, $S = 1$ and various values of $\xi _{a}$.

$\xi _{a}$	HAM result		BVP4c result		%Error = $\vert \frac{N.R - H.R}{N.R}\vert$	
	$F_{pres}$	$g'(1)$	$F_{pres}$	$g'(1)$	$F_{pres}$	$g'(1)$
0.1	19.2024	$-\,0.4561$	19.2023	$-\,0.4562$	$5.20771\times 10^{-6}$	$2.19202\times 10^{-3}$
0.5	6.9653	$-\,0.4723$	6.9652	$-\,0.4724$	$1.43571\times 10^{-4}$	$2.11775\times 10^{-3}$
1	$-\,34.0176$	$-\,0.5127$	$-34.0175$	$-\,0.5128$	$2.93966\times 10^{-6}$	$1.95008\times 10^{-3}$
1.5	$-\,268.29$	$-\,0.3399$	$-268.28$	$-\,0.3400$	$3.72745\times 10^{-4}$	$2.94204\times 10^{-3}$

**Table 8:** Computations for $f''({0})$, $-g'(0)$, $-h'(0)$, $-k'({0})$ and $-\theta '(0)$ with $\xi _{rad} = 0.2$, $\xi _{sq} = 0.2$, $\xi _{z} = 0.1$, $\xi _{\theta } = 1$, $\xi _{mag} = 0.5$, $\xi _{pr} = 2$, $\xi _{hm} = 1$, $\xi _{pm} = 1$, $\xi _{sm} = 0.5$, $\xi _{ec} = 2$, $S = 1$ and various values of $\xi _{vis}$.

$\xi _{vis}$	HAM result					BVP4c result				
	$f''({0})$	$-\,g'({0})$	$-\,h'({0})$	$-\,k'({0})$	$-\,\theta '({0})$	$f''({0})$	$-\,g'({0})$	$-\,h'({0})$	$-\,k'({0})$	$-\,\theta '({0})$
0.1	3.9532	1.8329	0.9267	0.7936	1.3785	3.9532	1.8329	0.9267	0.7936	1.3785
1	3.7789	0.3857	0.7837	1.037	1.3087	3.7789	0.3857	0.7837	1.037	1.3087
2	3.5985	0.0963	0.5893	1.4687	1.3097	3.5985	0.0963	0.5893	1.4687	1.3097
3	3.3789	0.0065	0.3784	1.7499	1.3001	3.3789	0.0065	0.3784	1.7499	1.3001

**Table 9:** Computations for $f''({0})$, $-\,g'(0)$, $-\,h'({0})$, $-\,k'({0})$ and $-\theta '(0)$ with $\xi _{rad} = 0.2$, $\xi _{sq} = 0.2$, $\xi _{z} = 0.1$, $\xi _{\theta } = 1$, $\xi _{pr} = 2$, $\xi _{hm} = 1$, $\xi _{pm} = 1$, $\xi _{sm} = 0.5$, $\xi _{ec} = 2$, $\xi _{vis} = 3$, $S = 1$ and various values of $\xi _{mag}$.

$\xi _{mag}$	HAM result					BVP4c result				
	$f''({0})$	$-\,g'({0})$	$-h'({0})$	$-\,k'({0})$	$-\,\theta '({0})$	$f''({0})$	$-g'({0})$	$-\,h'({0})$	$-k'({0})$	$-\,\theta '({0})$
0.5	3.1389	0.2598	0.7994	1.4829	3.2687	3.1389	0.2598	0.7994	1.4829	3.2687
1	3.1372	0.2598	0.5839	1.3799	1.3087	3.1372	0.2598	0.5839	1.3799	1.3087
2	3.1351	0.2599	0.4981	1.2911	1.3097	3.1351	0.2599	0.4981	1.2911	1.3097
3	3.1314	0.2599	0.2389	1.1999	1.3001	3.1314	0.2599	0.2389	1.1999	1.3001

**Table 10:** Computations for $f''({0})$, $g'(0)$, $h'(0)$ and $\theta '(0)$ with $\xi _{rad} = 0.5$, $\xi _{sq} = 1$, $\xi _{z} = 2$, $\xi _{\theta } = 1$, $\xi _{mag} = 3$, $\xi _{pr} = 0.2$, $\xi _{hm} = 1$, $\xi _{pm} = 0.2$, $\xi _{sm} = 1$, $\xi _{vis} = 3$, $S = 1$ and various values of $\xi _{ec}$.

$\xi _{ec}$	HAM Result					BVP4c result				
	$f''({0})$	$-\,g'({0})$	$-\,h'({0})$	$-\,\theta '({0})$	$-\,\Psi '({0})$	$f''({0})$	$-\,g'({0})$	$-\,h'({0})$	$-\,\theta '({0})$	$-\,\Psi '({0})$
1	3.0719	0.2981	0.1819	1.1371	1.0988	3.0719	0.2981	0.1819	1.1371	1.0988
2	3.0719	0.2981	0.1819	1.2811	1.1381	3.0719	0.2981	0.1819	1.2811	1.1381
3	3.0719	0.2981	0.1819	1.3101	1.2847	3.0719	0.2981	0.1819	1.3101	1.2847
4	3.0719	0.2981	0.1819	1.3919	1.3899	3.0719	0.2981	0.1819	1.3919	1.3899

**Table 11:** Computations for $f''({0})$, $-g'(0)$, $-h'(0)$, $-k'({0})$ and $-\theta '(0)$ with $\xi _{rad} = 0.2$, $\xi _{sq} = 0.2$, $\xi _{z} = 0.1$, $\xi _{\theta } = 1$, $\xi _{mag} = 1$, $\xi _{pr} = 2$, $\xi _{hm} = 1$, $\xi _{sm} = 0.5$, $\xi _{ec} = 2$, $\xi _{vis} = 3$, $S = 1$ and various values of $\xi _{pm}$.

$\xi _{pm}$	HAM Result					BVP4c result				
	$f''({0})$	$-\,g'({0})$	$-h'({0})$	$-k'({0})$	$-\,\theta '({0})$	$f''({0})$	$-\,g'({0})$	$-\,h'({0})$	$-\,k'({0})$	$-\,\theta '({0})$
0.1	3.1301	0.1299	0.8102	0.6989	1.1127	3.1301	0.1299	0.8102	0.6989	1.1127
0.5	3.1301	0.1299	0.8102	0.6989	1.2994	3.1301	0.1299	0.8102	0.6989	1.2994
1	3.1301	0.1299	0.8102	0.6989	1.3999	3.1301	0.1299	0.8102	0.6989	1.3999
2	3.1301	0.1299	0.8102	0.6989	1.5391	3.1301	0.1299	0.8102	0.6989	1.5391

**Table 12:** Computations for $f''({0})$, $g'(0)$, $h'(0)$, $-k'({0})$ and $\theta '(0)$ with $\xi _{rad} = 2$, $\xi _{sq} = 1$, $\xi _{z} = 0.1$, $\xi _{\theta } = 1$, $\xi _{mag} = 3$, $\xi _{pr} = 2$, $\xi _{hm} = 1$, $\xi _{pm} = 2$, $\xi _{ec} = 1$, $\xi _{vis} = 0.3$, $S = -0.5$ and various values of $\xi _{sm}$.

$\xi _{sm}$	HAM result					BVP4c result				
	$f''({0})$	$-g'({0})$	$-h'({0})$	$-k'({0})$	$-\theta '({0})$	$f''({0})$	$-g'({0})$	$-h'({0})$	$-k'({0})$	$-\theta '({0})$
0.1	3.9749	0.3769	0.3996	1.0929	0.9998	3.9749	0.3769	0.3996	1.0929	0.9998
0.5	3.9749	0.3769	0.3996	1.0929	1.0069	3.9749	0.3769	0.3996	1.0929	1.0069
1	3.9749	0.3769	0.3996	1.0929	1.0135	3.9749	0.3769	0.3996	1.0929	1.0135
1.5	3.9749	0.3769	0.3996	1.0929	1.0299	3.9749	0.3769	0.3996	1.0929	1.0299

**Figure 3:**
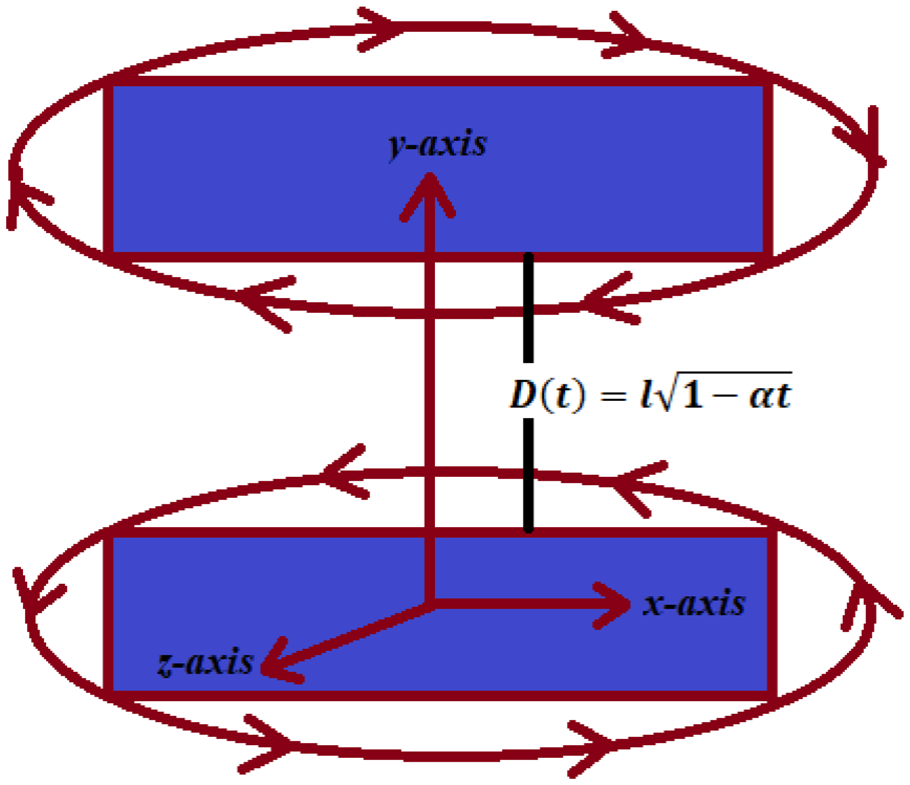
Geometry of the problem.

The greatest average residual-error of *f*(*eta*), *g*(*eta*), *h*(*eta*), *k*(*eta*), *ta*(*eta*), and *psi*(*eta*) using various approximating orders is shown in Fig. 3. In sub-figures it is evident that the error is virtually constantly decreased to $15{\rm th}$. Table 1 shows the overall residual error for various approximation orders, with fixed values, Table 2 shows comparison of HAM Results to numerical values for velocity field, heat and mass distributions variables. The BVP4*c* Results for $f''(0)$, $-g'(0)$, $-h'(0)$, $-k'(0)$, $-theta'(0)$, and $-Psi'(0)$ given in Table 3 provides further conformation of the authenticity for our results. Results converge almost on a $15{\rm th}$ approximation order. As the order of approximation is increased, the result converges to an exact solution. The results of HAM and BVP4*c* are compared in Tables 6, 7, 8, 9, 10, 11 and 12 for varied values of $\xi _{vis}, \xi _{rad}, \xi _{z}, \xi _{mag}, \xi _{ec}, \xi _{pm}, \xi _{sm}$ and fixed values of other associated parameters.

## Results and discussions

**Figure 4:**
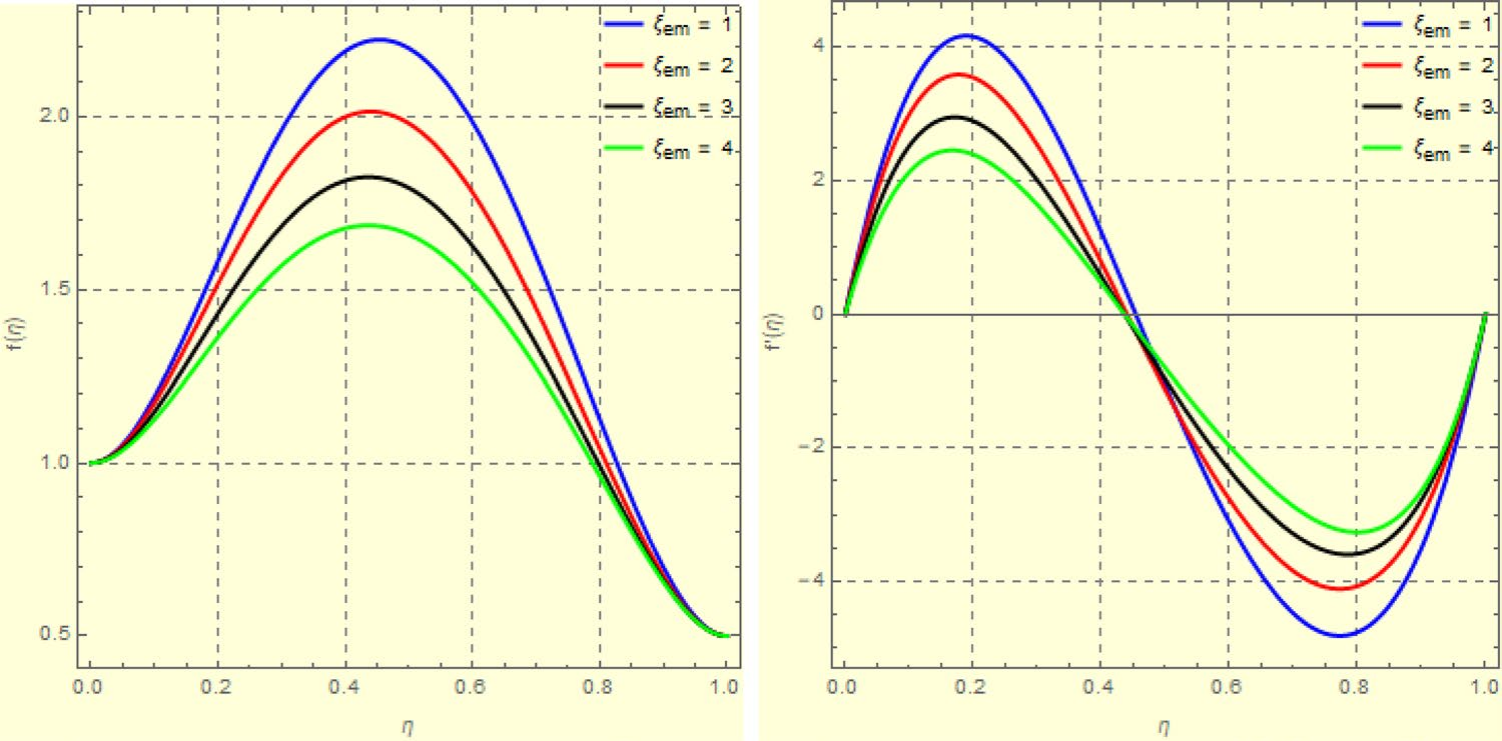
Impact of $\xi _{em}$ on $f({\eta} )$ and $f'({\eta} )$ for fixed values of $\xi _{a} = 0.3$, $\xi _{s} = 0.01$, $\xi _{z} = 5$, $\xi _{\theta } = 0.2$, $\xi _{pr} = 0.2$, $\xi _{hm} = 1$, $\xi _{pm} = 1$, $\xi _{sm} = 0.5$, $\xi _{ec} = 5$, $\xi _{v} = 0.1$ and $S = 0.5$.

**Figure 5:**
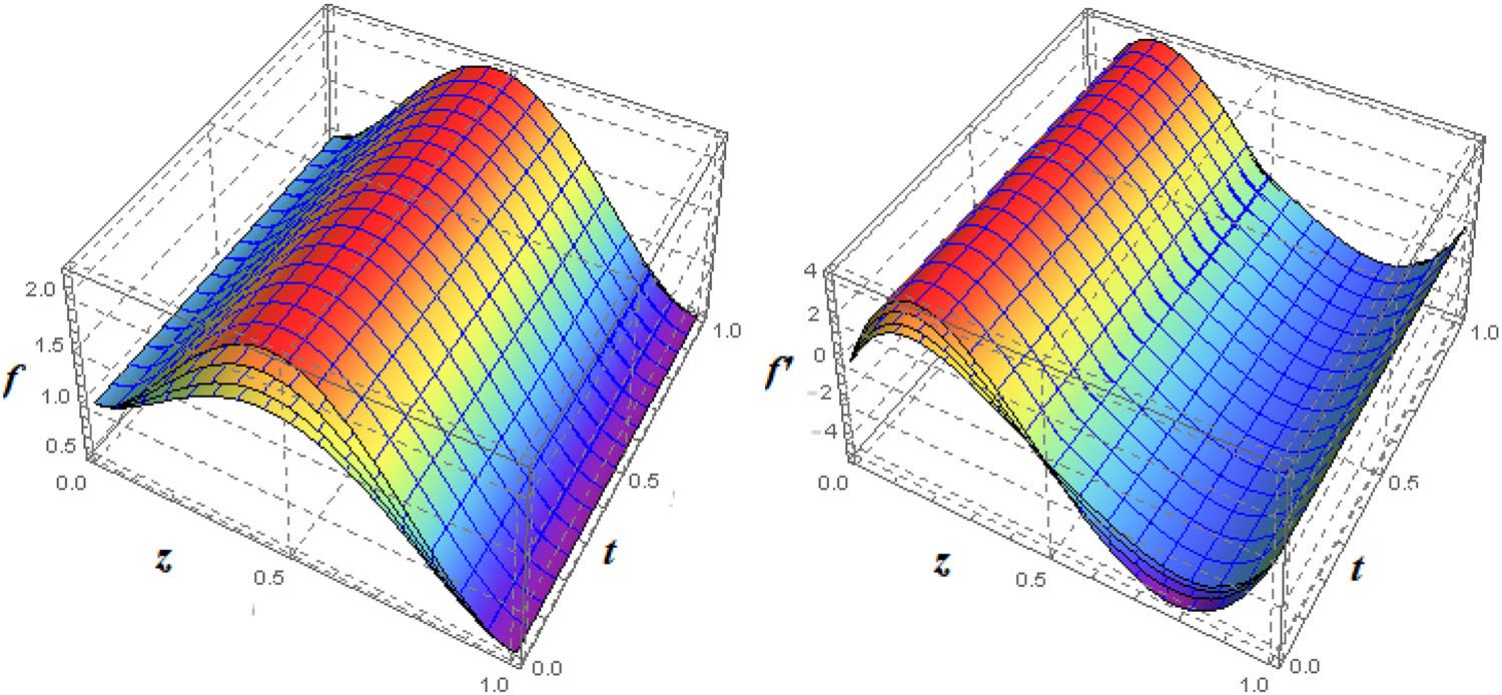
3*D*-view of the impact of $\xi _{em}$ on $f({\eta} )$ and $f'({\eta} )$ for fixed values of $\xi _{a} = 0.3$, $\xi _{s} = 0.01$, $\xi _{z} = 5$, $\xi _{\theta } = 0.2$, $\xi _{pr} = 0.2$, $\xi _{hm} = 1$, $\xi _{pm} = 1$, $\xi _{sm} = 0.5$, $\xi _{ec} = 5$, $\xi _{v} = 0.1$ and $S = 0.5$.

**Figure 6:**
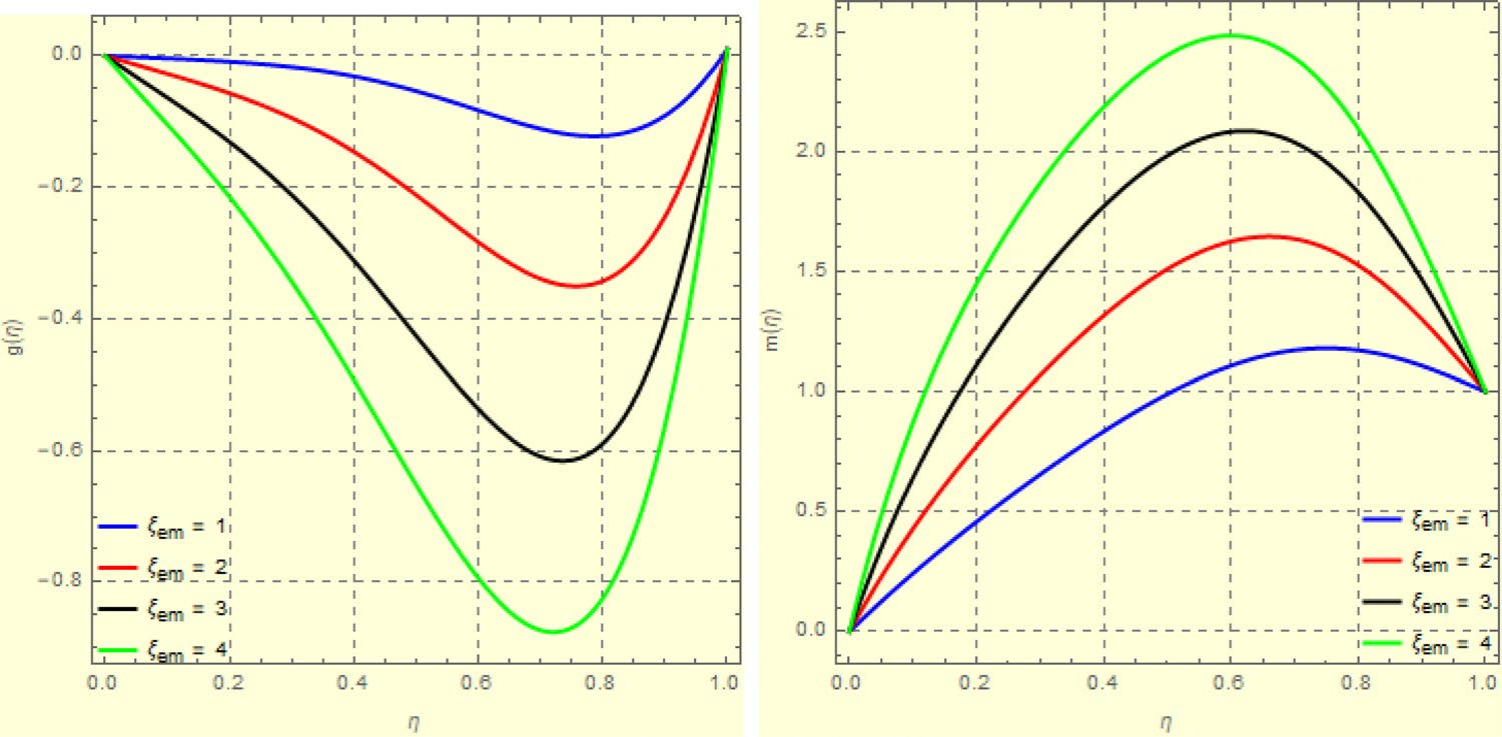
Impact of $\xi _{em}$ on $g({\eta} )$ and $h({\eta} )$ for fixed values of $\xi _{a} = 0.3$, $\xi _{s} = 0.01$, $\xi _{z} = 5$, $\xi _{\theta } = 0.2$, $\xi _{pr} = 0.2$, $\xi _{hm} = 1$, $\xi _{pm} = 1$, $\xi _{sm} = 0.5$, $\xi _{ec} = 5$, $\xi _{v} = 0.1$ and $S = 0.5$.

**Figure 7:**
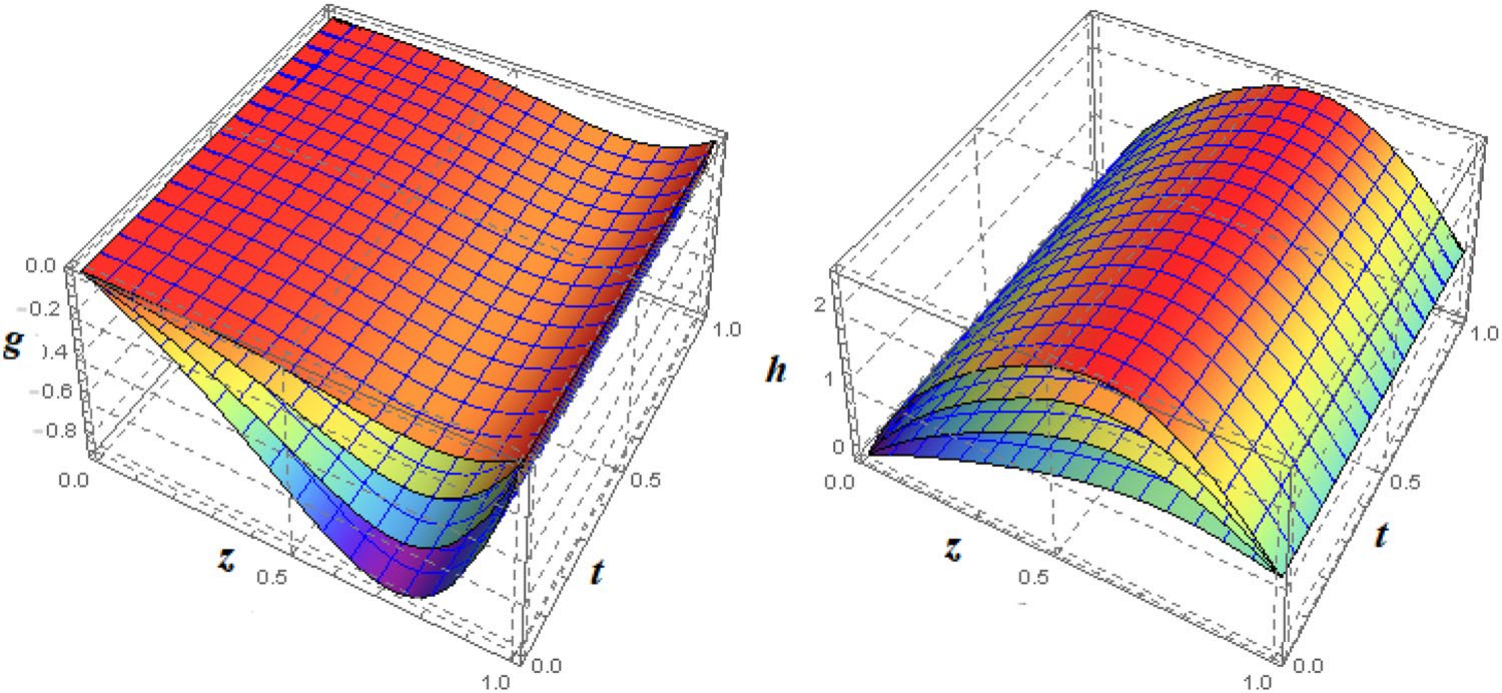
3*D*-view of the impact of $\xi _{em}$ on $g({\eta} )$ and $h({\eta} )$ for fixed values of $\xi _{a} = 0.3$, $\xi _{s} = 0.01$, $\xi _{z} = 5$, $\xi _{\theta } = 0.2$, $\xi _{pr} = 0.2$, $\xi _{hm} = 1$, $\xi _{pm} = 1$, $\xi _{sm} = 0.5$, $\xi _{ec} = 5$, $\xi _{v} = 0.1$ and $S = 0.5$.

**Figure 8:**
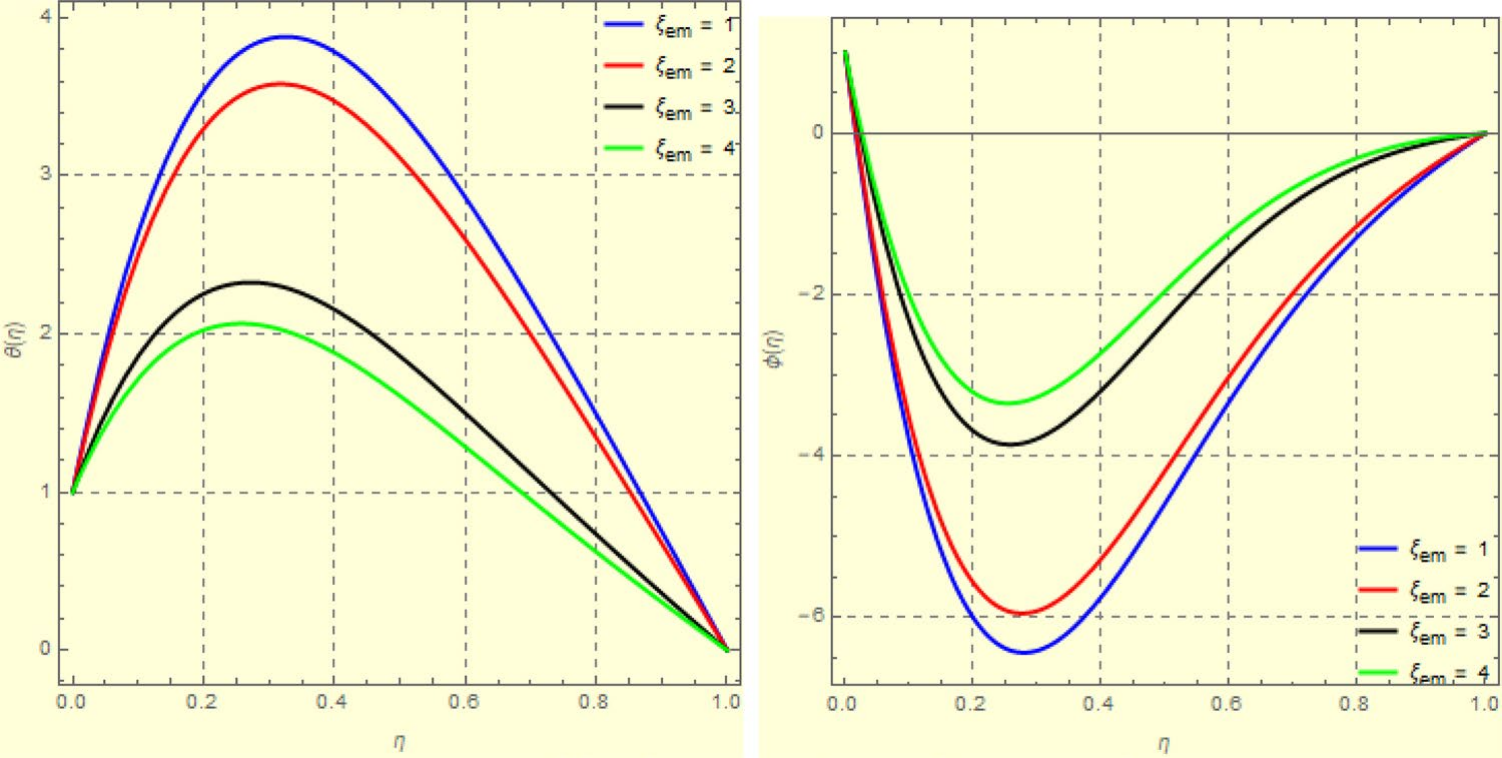
Impact of $\xi _{em}$ on $\theta ({\eta} )$ and $\phi ({\eta} )$ for fixed values of $\xi _{a} = 0.3$, $\xi _{s} = 0.01$, $\xi _{z} = 5$, $\xi _{\theta } = 0.2$, $\xi _{pr} = 0.2$, $\xi _{hm} = 1$, $\xi _{pm} = 1$, $\xi _{sm} = 0.5$, $\xi _{ec} = 5$, $\xi _{v} = 0.1$ and $S = 0.5$.

**Figure 9:**
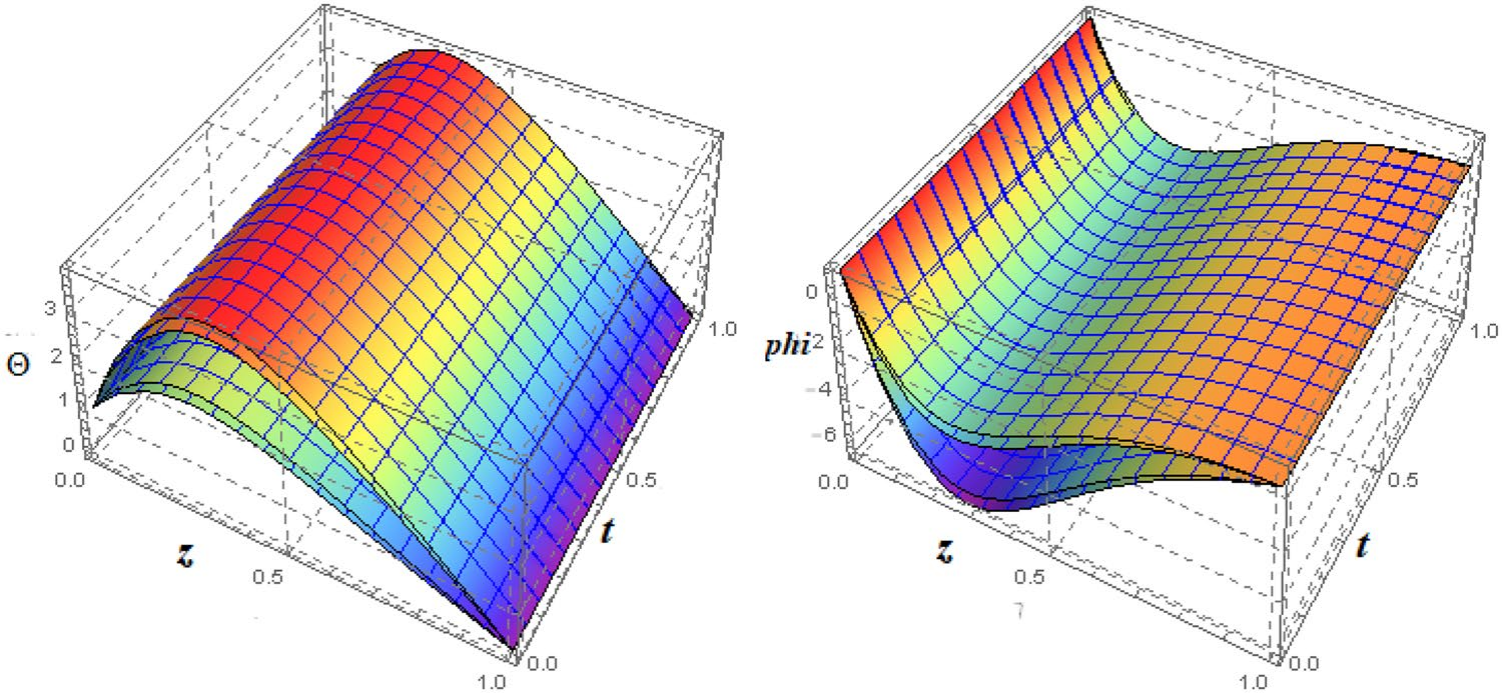
3*D*-view of the impact of $\xi _{em}$ on $\theta ({\eta} )$ and $\phi ({\eta} )$ for fixed values of $\xi _{a} = 0.3$, $\xi _{s} = 0.01$, $\xi _{z} = 5$, $\xi _{\theta } = 0.2$, $\xi _{pr} = 0.2$, $\xi _{hm} = 1$, $\xi _{pm} = 1$, $\xi _{sm} = 0.5$, $\xi _{ec} = 5$, $\xi _{v} = 0.1$ and $S = 0.5$.

**Figure 10:**
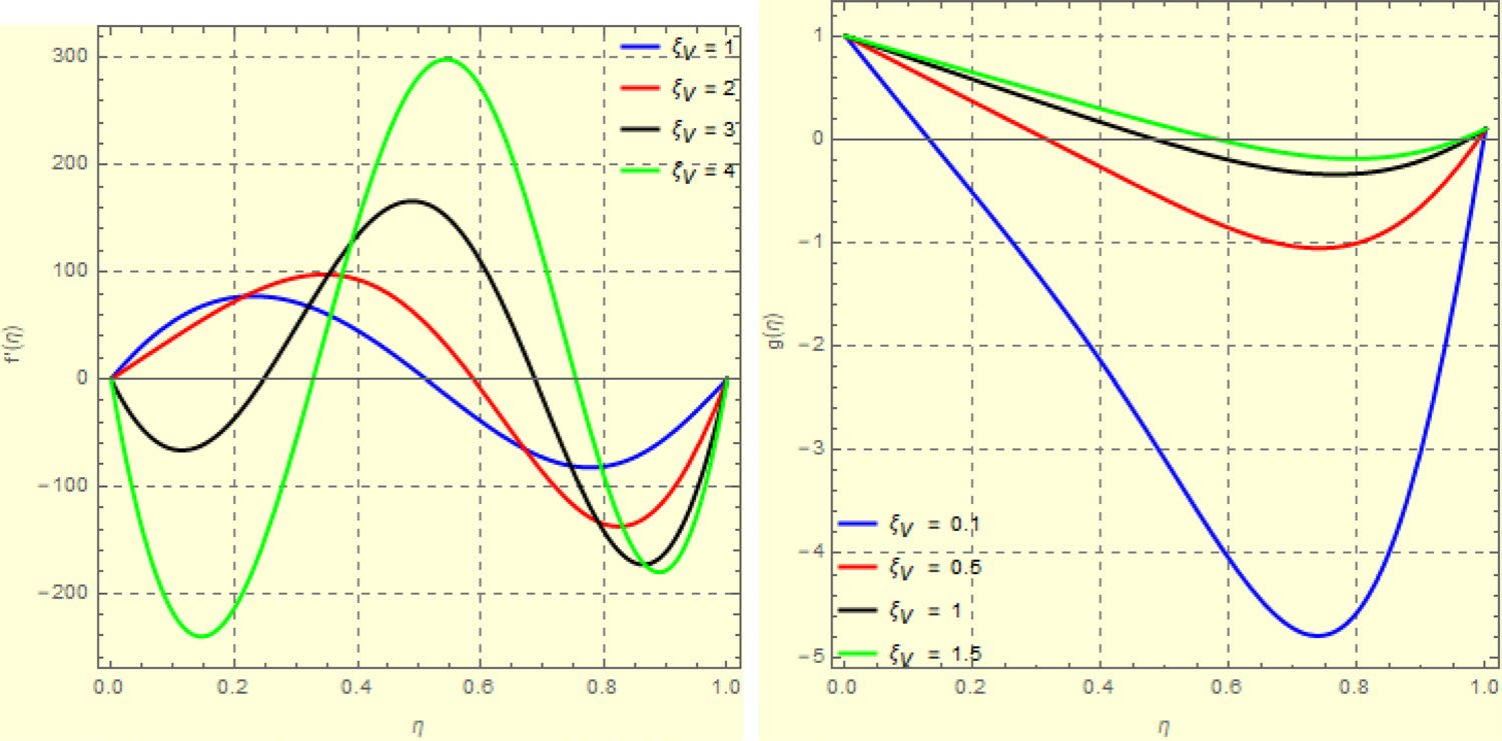
Impact of $\xi _{V}$ on $f'({\eta} )$ and $g({\eta} )$ for fixed values of $\xi _{a} = 1$, $\xi _{s} = 0.01$, $\xi _{z} = 3$, $\xi _{\theta } = 2$, $\xi _{em} = 0.5$, $\xi _{pr} = 0.2$, $\xi _{hm} = 1$, $\xi _{pm} = 1$, $\xi _{sm} = 1$, $\xi _{ec} = 0.3$ and $S = 1$.

**Figure 11:**
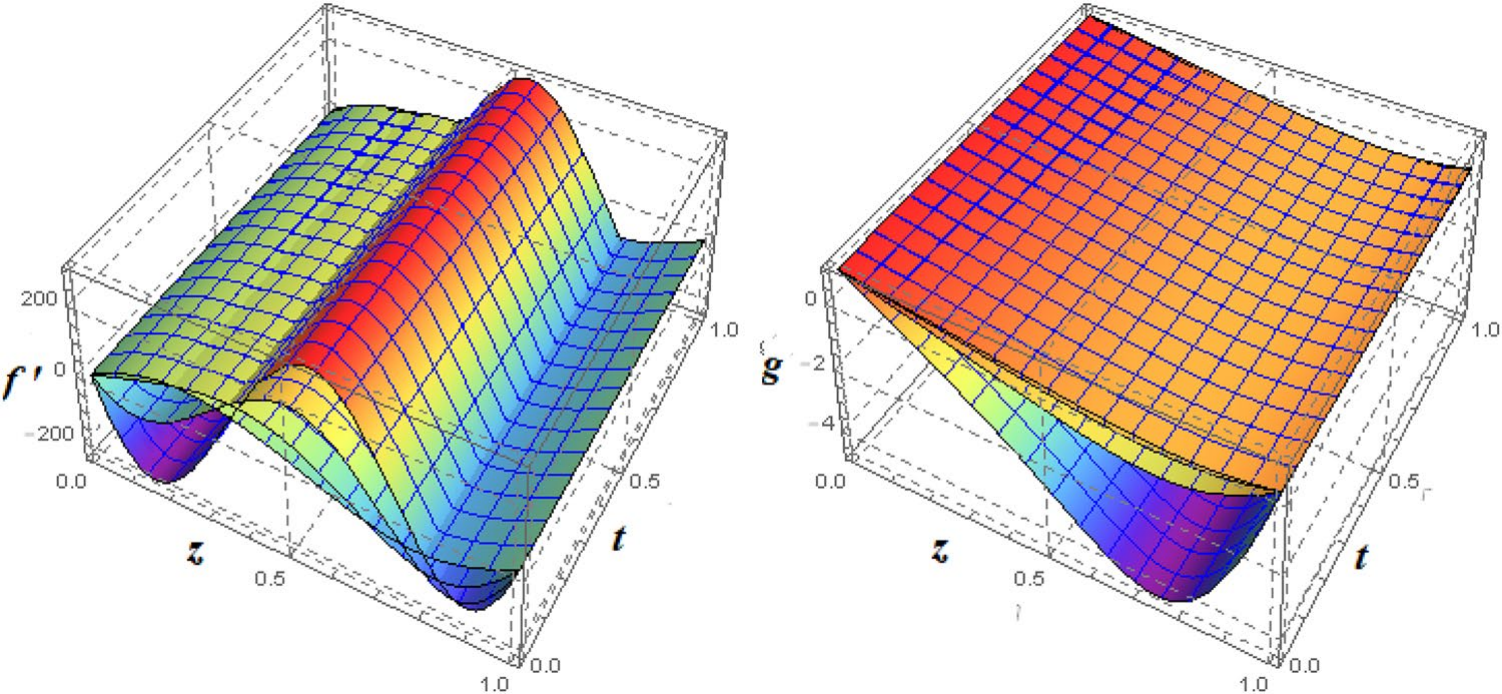
3*D*-view of the impact of $\xi _{V}$ on $f'({\eta} )$ and $g({\eta} )$ for fixed values of $\xi _{a} = 1$, $\xi _{s} = 0.01$, $\xi _{z} = 3$, $\xi _{\theta } = 2$, $\xi _{em} = 0.5$, $\xi _{pr} = 0.2$, $\xi _{hm} = 1$, $\xi _{pm} = 1$, $\xi _{sm} = 1$, $\xi _{ec} = 0.3$ and $S = 1$.

**Figure 12:**
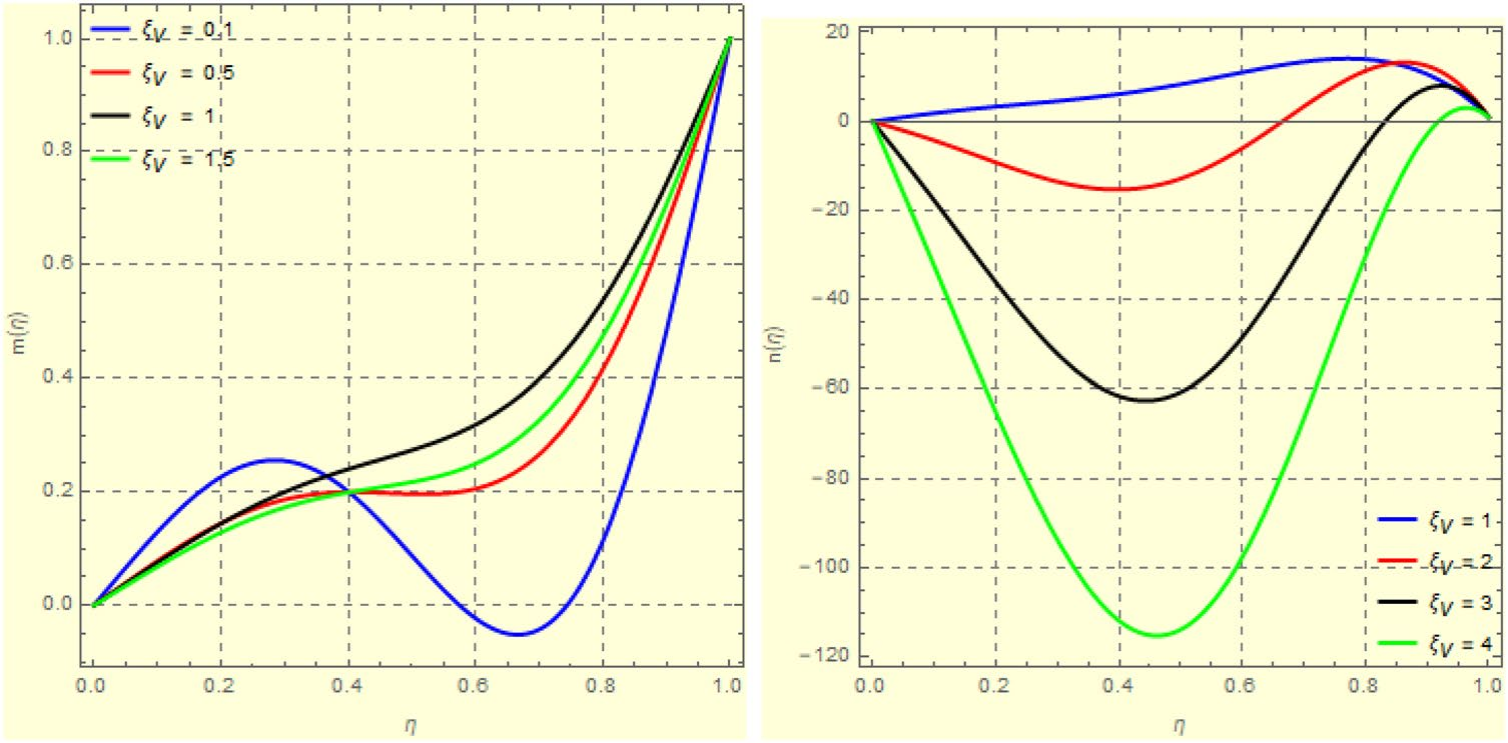
Impact of $\xi _{V}$ on $h({\eta} )$ and $k({\eta} )$ for fixed values of $\xi _{a} = 1$, $\xi _{s} = 0.01$, $\xi _{z} = 3$, $\xi _{\theta } = 2$, $\xi _{em} = 0.1$, $\xi _{pr} = 2$, $\xi _{hm} = 1$, $\xi _{pm} = 1$, $\xi _{sm} = 1$, $\xi _{ec} = 0.3$ and $S = 1$.

**Figure 13:**
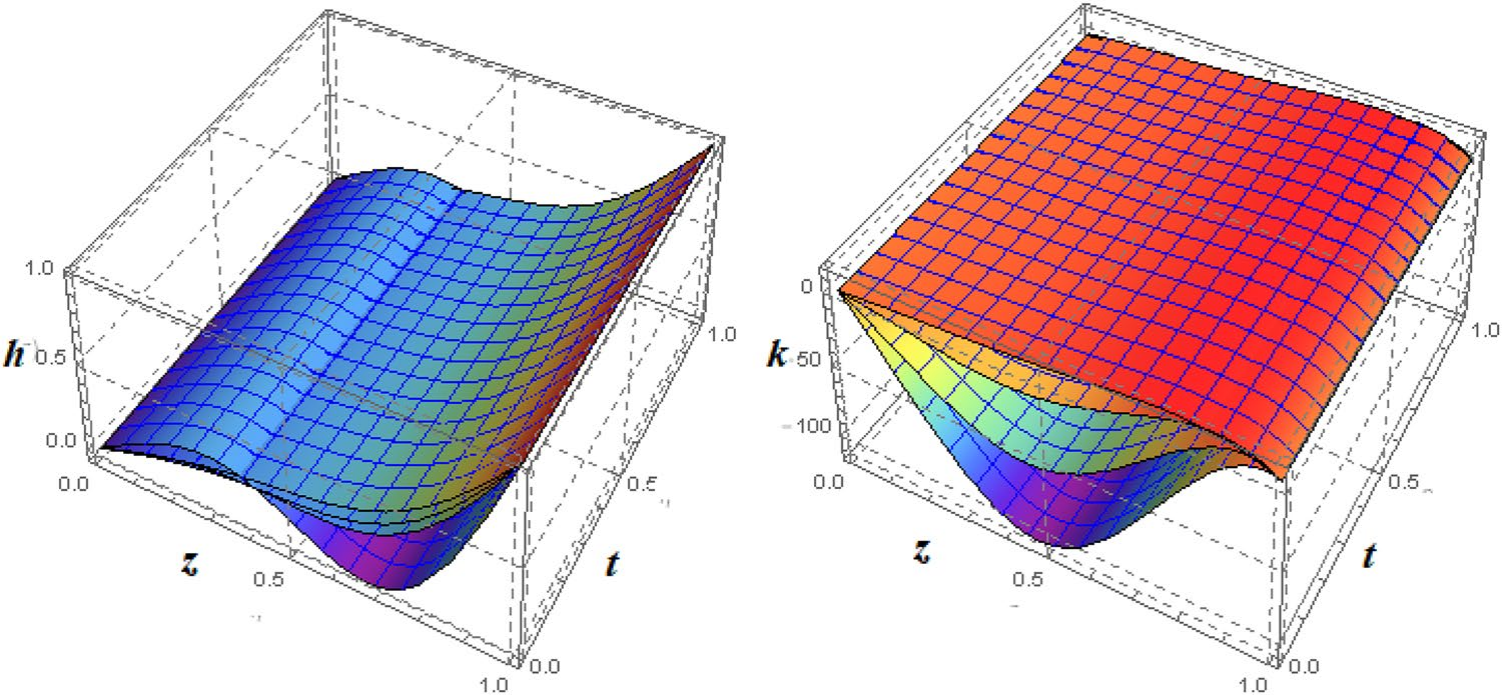
3*D*-view of the impact of $\xi _{V}$ on $h({\eta} )$ and $k({\eta} )$ for fixed values of $\xi _{a} = 1$, $\xi _{s} = 0.01$, $\xi _{z} = 3$, $\xi _{\theta } = 2$, $\xi _{em} = 0.1$, $\xi _{pr} = 2$, $\xi _{hm} = 1$, $\xi _{pm} = 1$, $\xi _{sm} = 1$, $\xi _{ec} = 0.3$ and $S = 1$.

**Figure 14:**
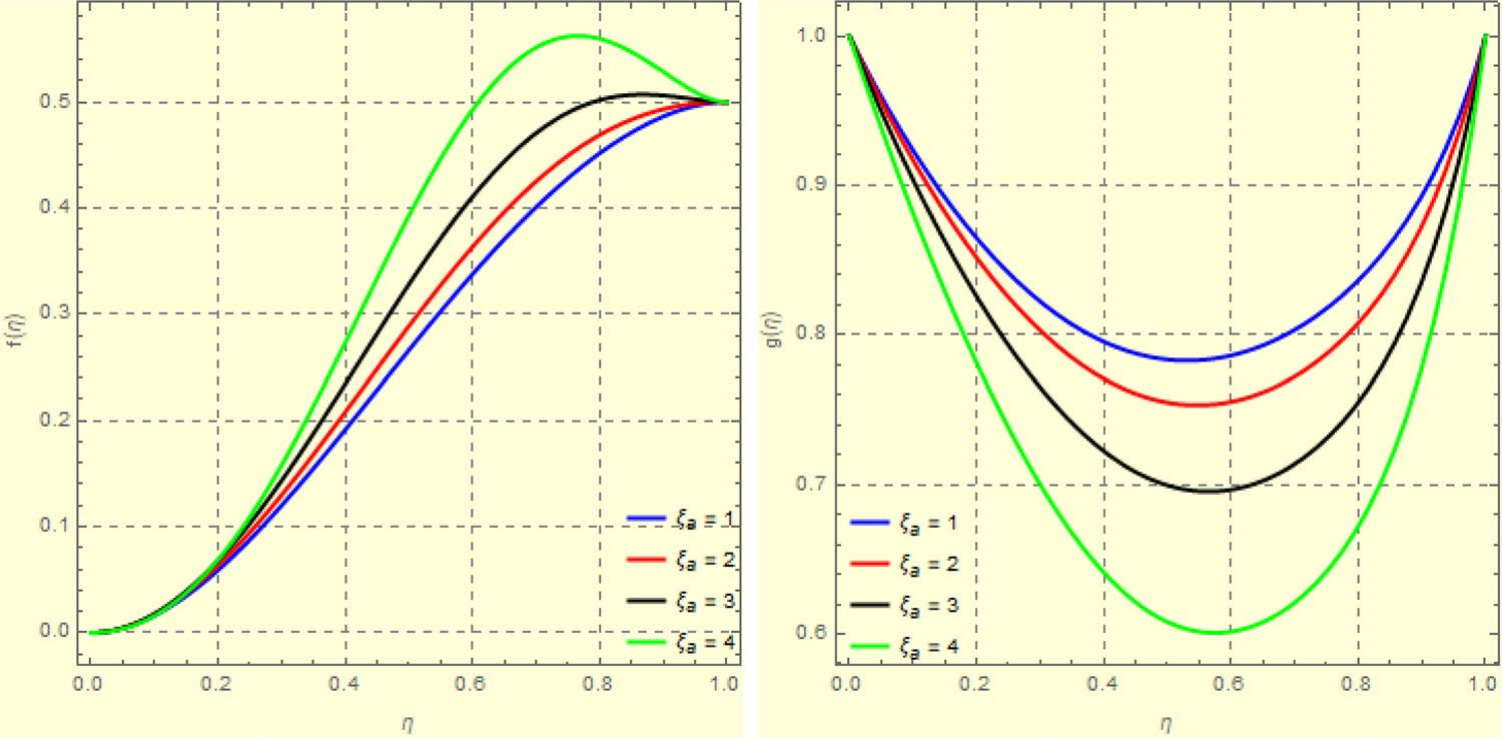
Impact of $\xi _{a}$ on $f({\eta} )$ and $g({\eta} )$ for fixed values of $\xi _{s} = 1$, $\xi _{z} = 5$, $\xi _{\theta } = 0.2$, $\xi _{em} = 5$, $\xi _{pr} = 1$, $\xi _{hm} = 2$, $\xi _{pm} = 1$, $\xi _{sm} = 0.5$, $\xi _{ec} = 3$, $\xi _{v} = 0.5$ and $S = 0.5$.

**Figure 15:**
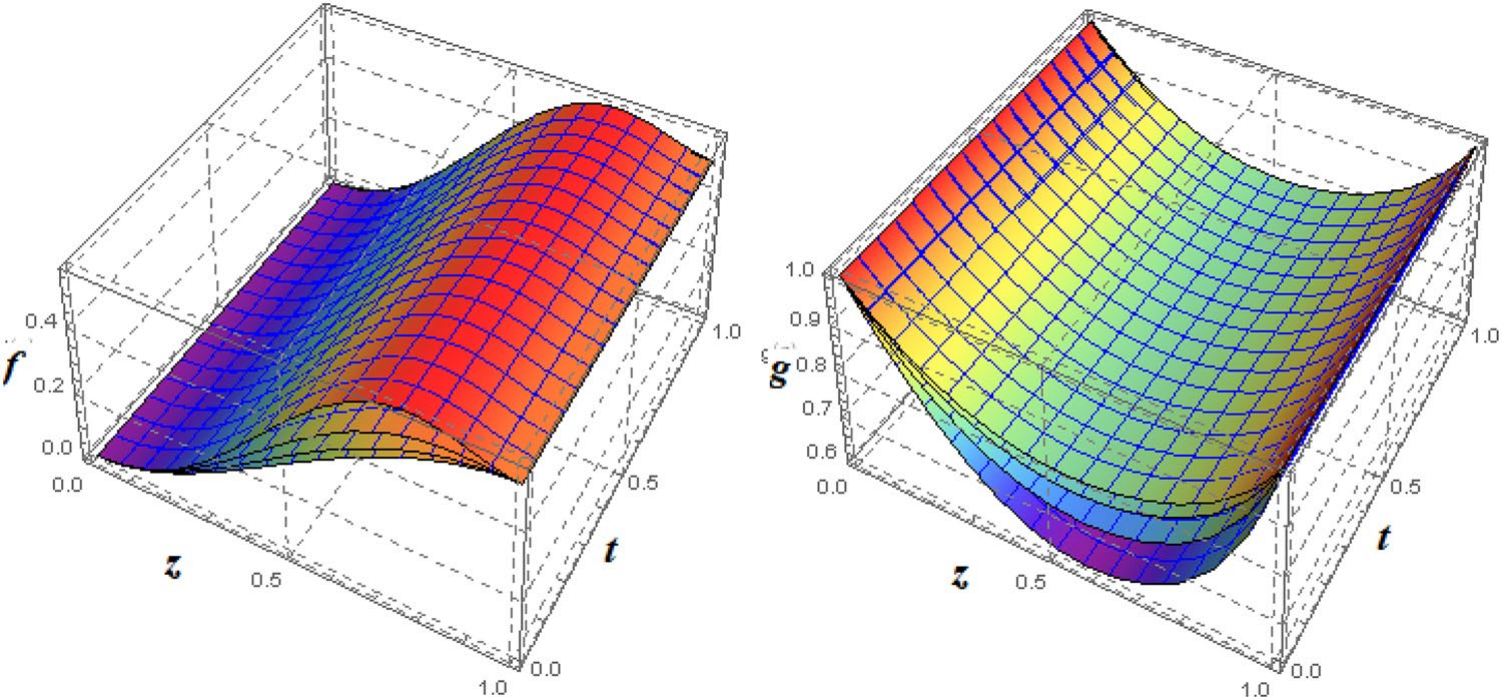
3*D*-view of the impact $\xi _{a}$ on $f({\eta} )$ and $g({\eta} )$ for fixed values of $\xi _{s} = 1$, $\xi _{z} = 5$, $\xi _{\theta } = 0.2$, $\xi _{em} = 5$, $\xi _{pr} = 1$, $\xi _{hm} = 2$, $\xi _{pm} = 1$, $\xi _{sm} = 0.5$, $\xi _{ec} = 3$, $\xi _{v} = 0.5$ and $S = 0.5$.

**Figure 16:**
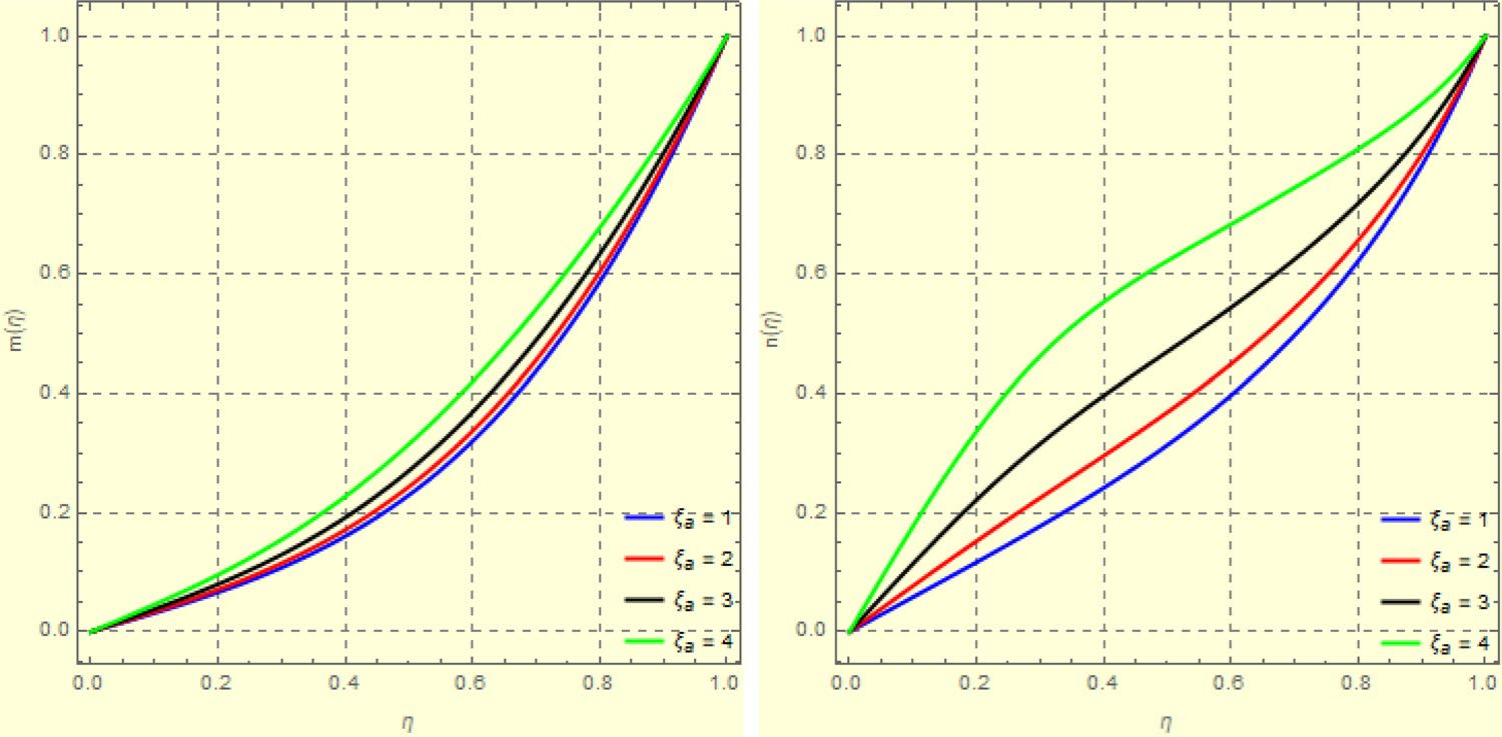
Impact of $\xi _{a}$ on $h({\eta} )$ and $k({\eta} )$ for fixed values of $\xi _{s} = 1$, $\xi _{z} = 5$, $\xi _{\theta } = 0.2$, $\xi _{em} = 5$, $\xi _{pr} = 1$, $\xi _{hm} = 2$, $\xi _{pm} = 1$, $\xi _{sm} = 0.5$, $\xi _{ec} = 3$, $\xi _{v} = 0.5$ and $S = 0.5$.

**Figure 17:**
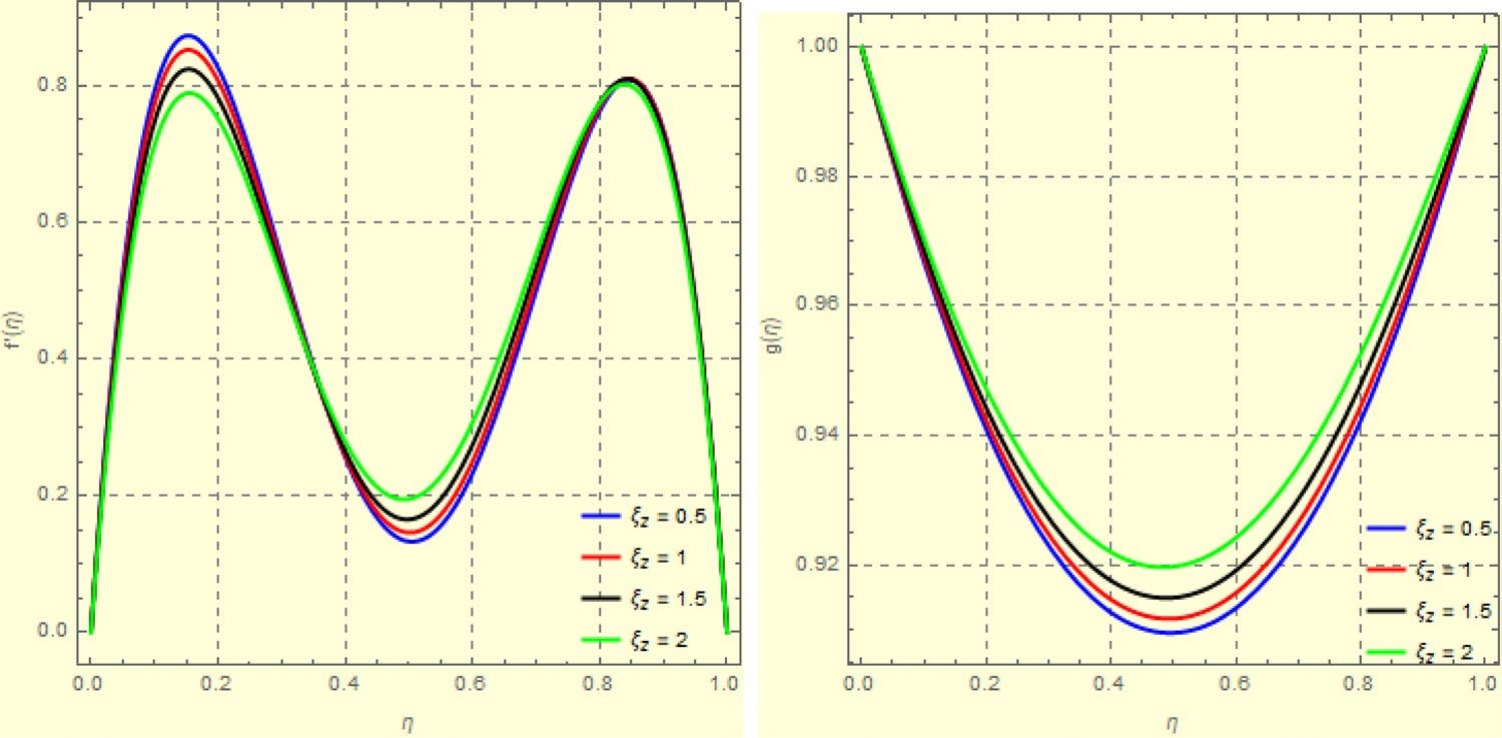
Impactof $\xi _{z}$ on $f'({\eta} )$ and $g({\eta} )$ for fixed values of $\xi _{a} = 1$, $\xi _{s} = 0.1$, $\xi _{\theta } = 0.1$, $\xi _{em} = 1$, $\xi _{pr} = 0.1$, $\xi _{hm} = 10$, $\xi _{pm} = 1$, $\xi _{sm} = 1$, $\xi _{ec} = 0.1$, $\xi _{v} = 0.1$ and $S = 1$.

**Figure 18:**
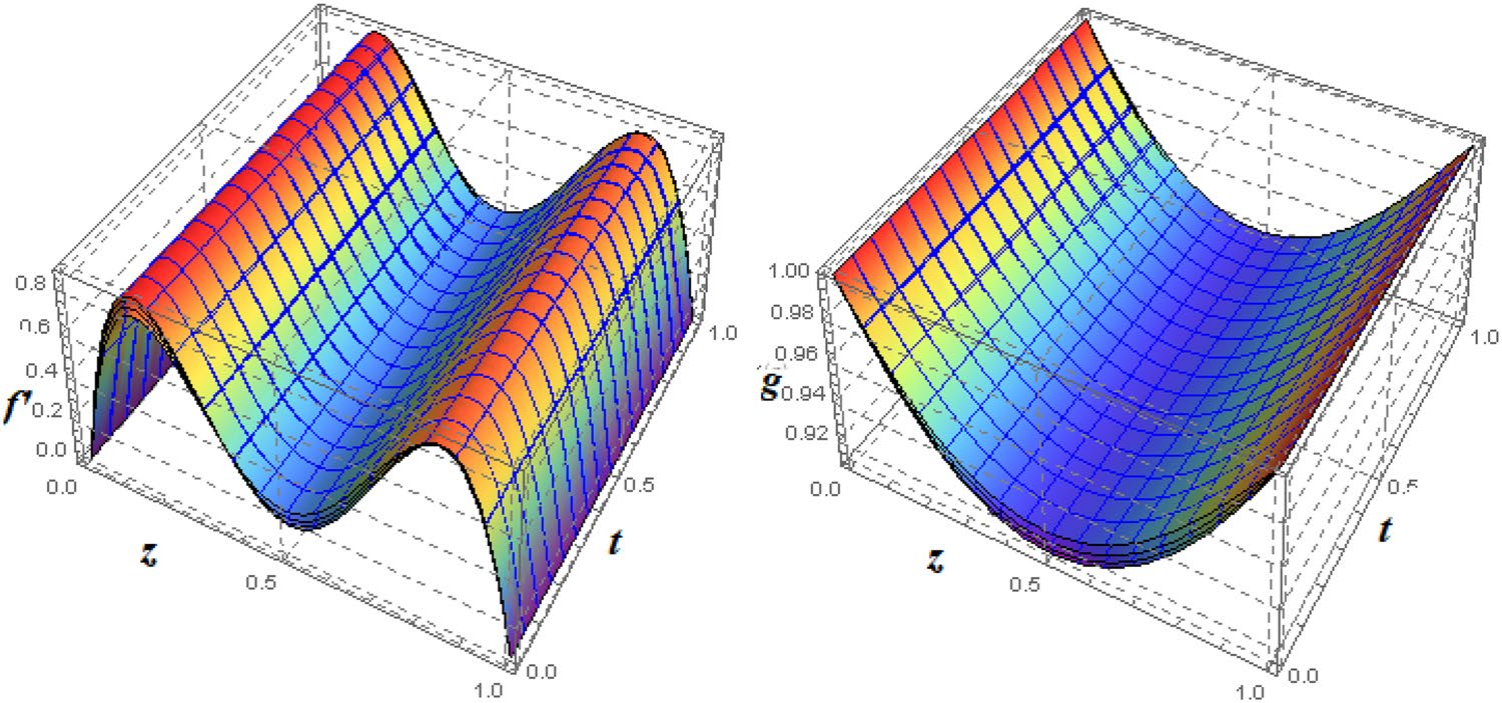
3*D*-view of the Impact $\xi _{z}$ on $f({\eta} )$, $f'({\eta} )$ for fixed values of $\xi _{a} = 1$, $\xi _{s} = 0.1$, $\xi _{\theta } = 0.1$, $\xi _{em} = 1$, $\xi _{pr} = 0.1$, $\xi _{hm} = 10$, $\xi _{pm} = 1$, $\xi _{sm} = 1$, $\xi _{ec} = 0.1$, $\xi _{v} = 0.1$ and $S = 1$.

**Figure 19:**
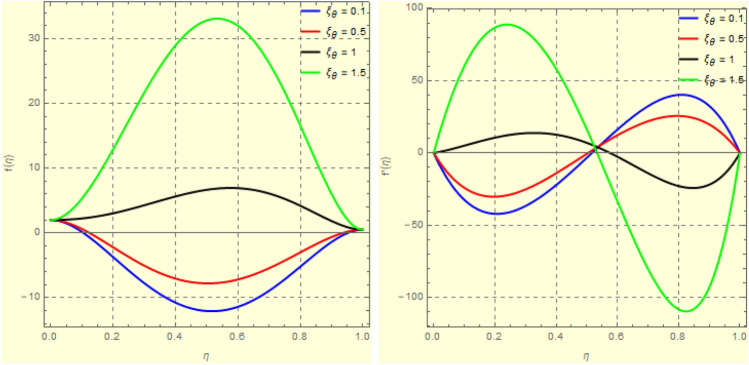
Impact of $\xi _{em}$ of $f({\eta} )$ and $f'({\eta} )$ for fixed values of $\xi _{a} = 2$, $\xi _{s} = 0.1$, $\xi _{z} = 0.5$, $\xi _{em} = 1$, $\xi _{pr} = 1$, $\xi _{hm} = 10$, $\xi _{pm} = 1$, $\xi _{sm} = 1$, $\xi _{ec} = 0.1$, $\xi _{v} = 0.1$ and $S = 1$.

**Figure 20:**
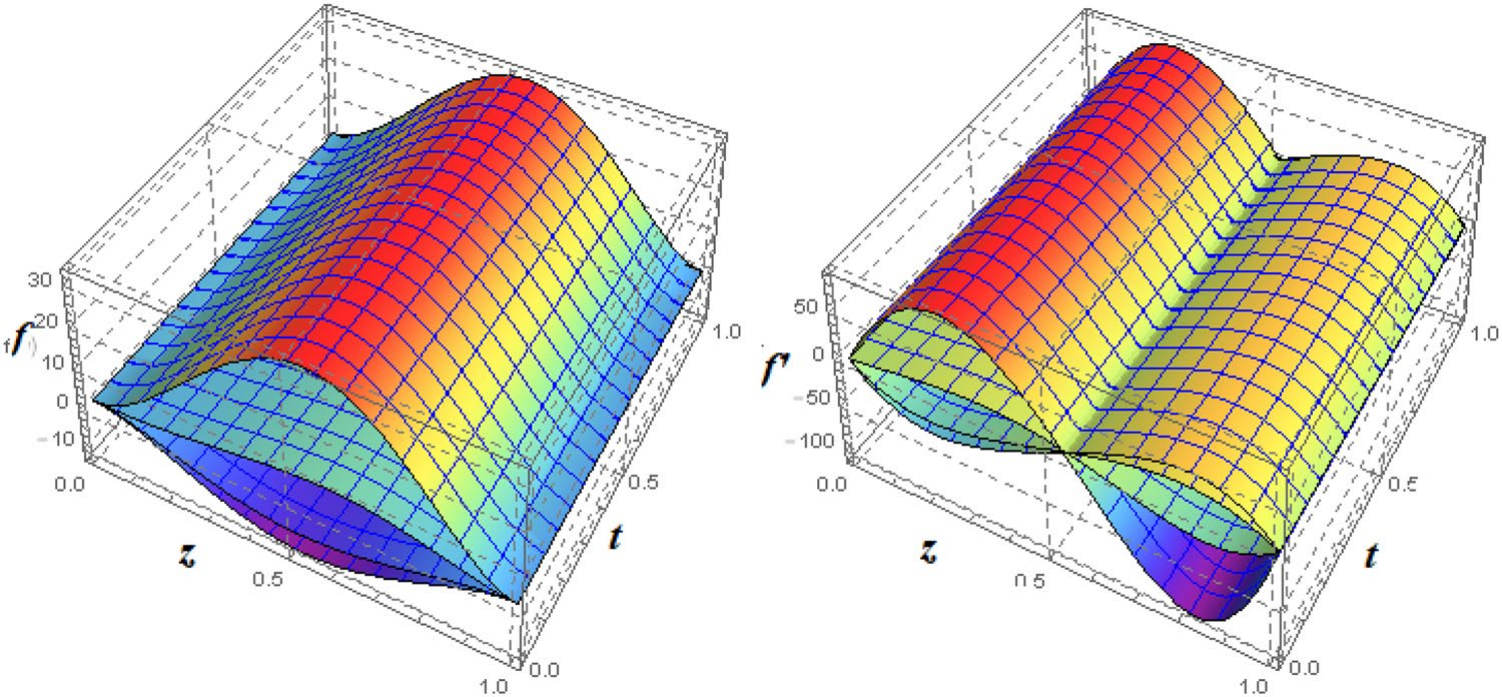
3*D*-view of the impact $\xi _{a}$ $f({\eta} )$ and $f'({\eta} )$ for fixed values of $\xi _{a} = 2$, $\xi _{s} = 0.1$, $\xi _{z} = 0.5$, $\xi _{em} = 1$, $\xi _{pr} = 1$, $\xi _{hm} = 10$, $\xi _{pm} = 1$, $\xi _{sm} = 1$, $\xi _{ec} = 0.1$, $\xi _{v} = 0.1$ and $S = 1$.

**Figure 21:**
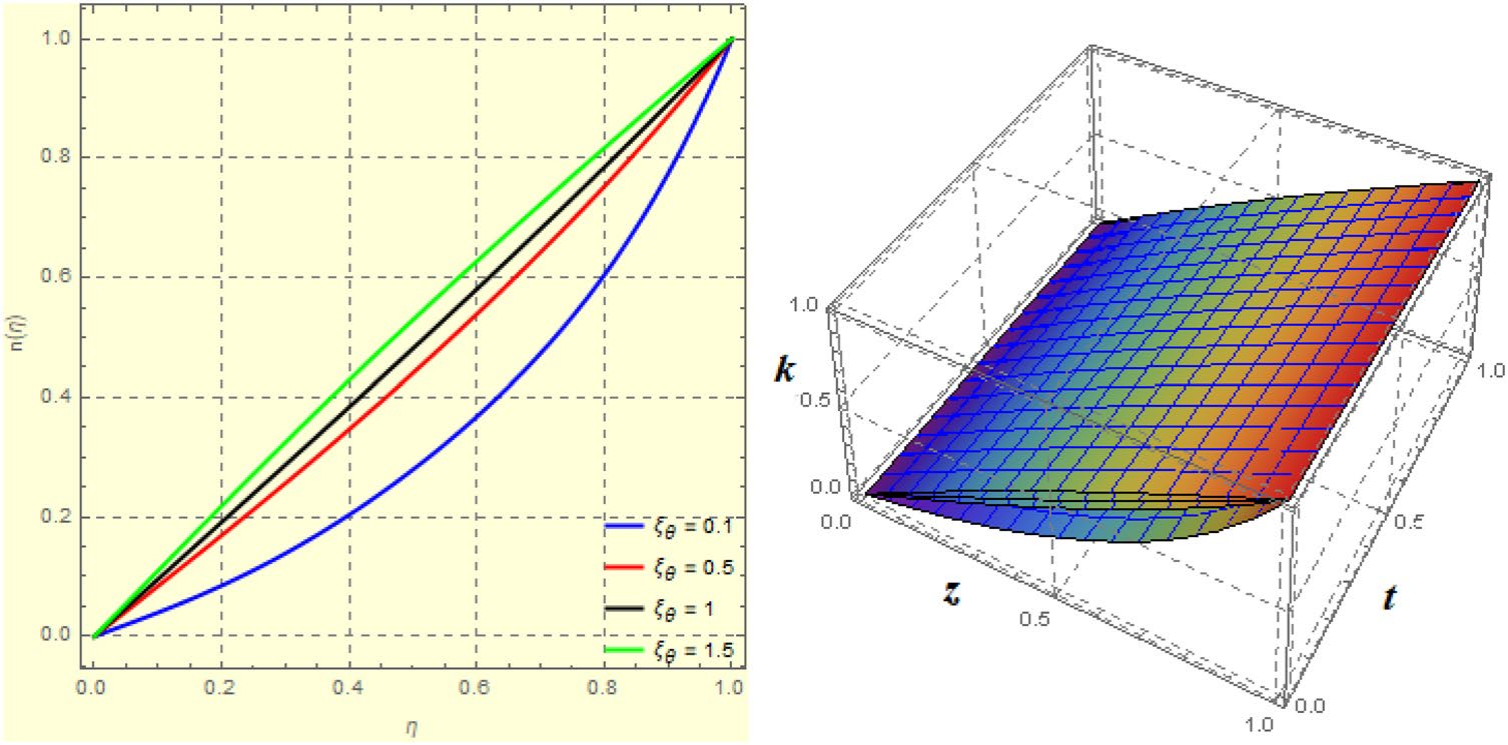
Impact of $\xi _{\theta }$ on $k({\eta} )$ for fixed values of $\xi _{a} = 2$, $\xi _{s} = 0.1$, $\xi _{z} = 0.5$, $\xi _{em} = 1$, $\xi _{pr} = 1$, $\xi _{hm} = 10$, $\xi _{pm} = 1$, $\xi _{sm} = 1$, $\xi _{ec} = 0.1$, $\xi _{v} = 0.1$ and $S = 1$.

**Figure 22:**
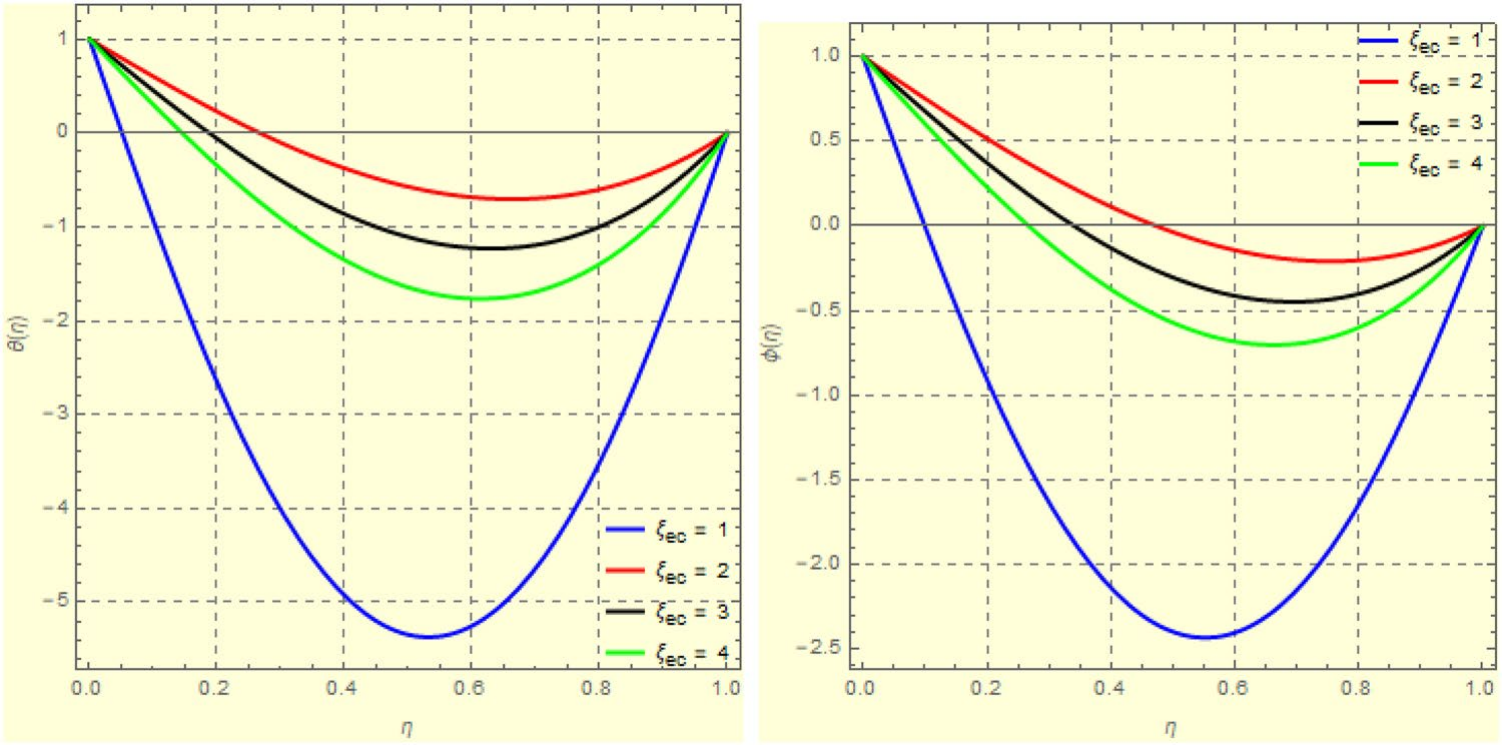
Impact of $\xi _{ec}$ on $\theta ({\eta} )$ and $\phi ({\eta} )$ for fixed values of $\xi _{a} = 0.2$, $\xi _{s} = 0.2$, $\xi _{z} = 0.1$, $\xi _{\theta } = 1$, $\xi _{em} = 0.5$, $\xi _{pr} = 10$, $\xi _{hm} = 10$, $\xi _{pm} = 1$, $\xi _{sm} = 0.5$, $\xi _{v} = 1$ and $S = -1$.

**Figure 23:**
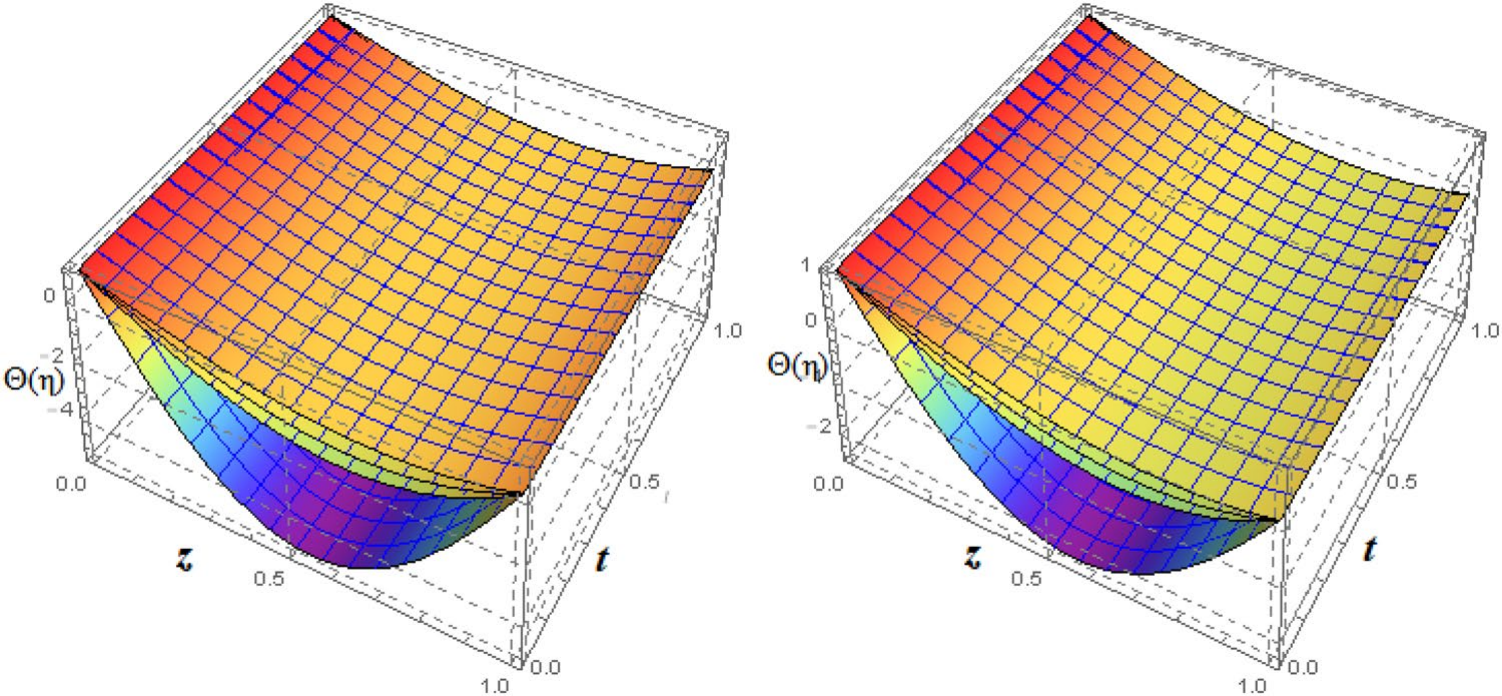
3*D*-view of $\theta ({\eta} )$ and $\phi ({\eta} )$ for fixed values of $\xi _{a} = 0.2$, $\xi _{s} = 0.2$, $\xi _{z} = 0.1$, $\xi _{\theta } = 1$, $\xi _{em} = 0.5$, $\xi _{pr} = 10$, $\xi _{hm} = 10$, $\xi _{pm} = 1$, $\xi _{sm} = 0.5$, $\xi _{v} = 1$ and $S = -1$.

To examine and analyse how the magnetic field and squeezing phenomena affect flow rate in the presence of MFD temperature, optimise the system’s heating/cooling process, lessen fluid turbulence, and scale up flow tracers. using the laminar, unstable, and incompressible MFD viscous fluid flow produced by compressing plates under the influence of no-slip velocity and convective surface boundary conditions. to look into the flow for mass and heat transfer and to offer an analytical and numerical study of it. For the cases of rotating and compressing plates, the influence of the different flow parameters is addressed graphically. The influence of the flow parameters is given for the vertical velocity component $f'({\eta} )$, horizontal velocity compnent $f({\eta} )$, magnetic field components and temperature $\theta ({\eta} )$ and mass transfer $\Psi ({\eta} )$ variations, respectively. In this section the impact of rotational parameter $\xi _{rot}$, squeeze Reynold number $\xi _{sq}$, Prandtl number $\xi _{pr}$, Hartman number $\xi _{hm}$ and all other parameters are respectively analyzed and discussed in detail. Figures 4, 5, 6, 7, 8, 9, 10, 11, 12, 13, 14, 15, 16, 17, 18, 19, 20, 21, 22 and 23 and Tables 5, 6, 7, 8, 9, 10, 11 and 12 depict effect of the natural parameters that appear during mathematical modelling of this problem. Dimensionless parameters are analysed for $\xi _{vis}$, $\xi _{a}$, $\xi _{z}$, $\xi _{\theta }$, $\xi _{mag}$, $\xi _{pm}$, $\xi _{sm}$ and $\xi _{ec}$ on the velocity field components $f'({\eta} )$, $g({\eta} )$, $f({\eta} )$, components of the magnetic field $h({\eta} )$, $k({\eta} )$ and heat/mass distributions $\theta ({\eta} )$, $\Psi ({\eta} )$ respectively. To model physically realistic flows by HAM , representative values are used.

Figures 4 and 5 depicts the effect of $\xi _{mag} = \xi _{sq}\xi _{bt}$ on $f({\eta} )$ and $f'({\eta} )$. The ratio of the fluid flux to magnetic diffusivity is described by $\xi _{mag}$. As a result, along streamlines this parameter is critical in evaluating magnetic field diffusion^15^. As seen in Fig. 4, a decrease in the magnetic diffusion enhances the axial and radial velocity distributions neat the lower plate. The minimum value of $f({\eta} )$ and $f'({\eta} )$ appears near the lower plate. As fluid travels from the central region toward upper plate, the influence of $\xi (mag)$ on $f'({\eta} )$ and $f({\eta} )$ decreases due to the decrease in the magnetic flux. Figure 5 is made to depict the 3*D*-view of the velocity components. The influence of $\xi _{mag}$ on $g({\eta} )$ and $h({\eta} )$ is shown in Figs. 6 and 7 . As seen in Fig. 6a, the minimum value of $g({\eta} )$ appears near the upper plate due to the resistance of magnetic diffusion to the horizontal and vertical velocities, similarly in Fig. 6b, an increase in fluid flux or a reduction in magnetic diffusivity has a clear effect on $h({\eta} )$. Figure 7 show the 3*D*-view of $g({\eta} )$ and $h({\eta} )$ for fixed values of other parameters. The effect of $\xi _{mag}$ is also investigated for $\theta ({\eta} )$ and concentration distribution. For this purpose, Figs. 8 and 9 are drawn. A decrease in the magnetic diffusion enhances the fluid viscosity will decrease $\theta ({\eta} )$ near the upper plate, similarly $\Psi ({\eta} )$ both decrease strongly. Figure 9 is made to to observed this phenomenon in a 3*D*-view.

The effects of MFD viscosity $\xi _{vis}$ on $f'({\eta} )$ and $g({\eta} )$ are shown in Figs. 10 and 11. A higher viscosity coefficient means that a fluid has more flow resistance. Figure 10 shows that the flow of fluid near the plates reduces in the horizontal direction as the fluid’s viscosity increases because of an increase in the fluid’s flow resistance. It’s also important to note that fluid density begins to rise in the fluid domain’s centre area as it moves in the direction of the top plate. An increase in $\xi _{vis}$ has an impact on azimuthal velocity, as seen in Fig. 11b. The highest value of $\xi _{vis} = 4$ has the greatest drop in $g({\eta} )$ at the upper disc. Figure 11 shows this phenomena in a 3*D*-dimensional view. Investigations on the magnetic field’s impact of MFD viscosity are also conducted. The $h({\eta} )$ and $k({\eta} )$ can be impacted by changes in MFD viscosity, as would be predicted. Figure 12 illustrates how $h({\eta} )$ and $k({\eta} )$ both display the opposing action of $\xi _{vis}$. This phenomena is depicted in Fig. 13 in a 3*D*-dimensional form.

One of the most significant physical parameter is the rotational Reynolds parameter. The effect of the rotational Reynolds number $$\xi _{a} = \frac{\Omega _{l}l^{2}}{\xi _{vis}}$$ could be observed in Figs. 13 and 14. A rise in $$\xi _{a}$$ indicates either an increase in the lower plate angular velocity or a reduction in fluid viscosity. Expanding the value of $$\xi _{a}$$, in vicinity of plates, the axial velocity reduces. This is because of the rise of angular velocity of the lower plate (both plates rotate in the same direction as $$S = 0.5 > 0)$$ pushes fluid in a radial direction, reducing fluid movement in the axial direction near the plates. As the fluid flows into the central region, the rotational strength of the plates decreases, and the velocity begins to increase in the axial direction after the central region, as seen in Fig. 14b. Figure 15 is made to observed this phenomenon in a 3*D*-view. Figure 16 depicts the effect of $$\xi _{a}$$ on the axial as well as azimuthal portion of the induced magnetic field, $$h({\eta} )$$. Clearly increase in $$\xi _{a}$$ strengthens $$h({\eta} )$$.

The impact of the dimensionless strength of the axial magnetic field $\xi _{z}$ is depicted in Figs 17 and 18. The influence of $\xi _{z}$ on $f'({\eta} )$ and $g({\eta} )$ near plates has been seen to be rather small, but it becomes noticeable when fluid moves into the higher plate. $f'({\eta} )$ starts to rise at the fluid domain’s centre, then falls as it gets closer to the top disc. Similar to this, $g({\eta} )$ drops as $\xi _{z}$ grows. The greatest decrease is visible towards the centre of the fluid domain. Figure 18 is designed to show the effect of $\xi _{z}$ from a three-dimensional perspective. Figures 19 and 21 show how $\xi _{\theta }$ has an effect on $f({\eta} )$, $f'({\eta} )$, and $k({\eta} )$. The applied magnetic field’s dimensionless azimuthal amplitude is $\xi _{\theta }$. It is important to keep in mind that rising $\xi _{\theta }$ raises both $f({\eta} )$ and $f'({\eta} )$. This is due to the fact that at the top disc, where the angular velocities of the lower plate’s $Omega_l$ and $Omega_u$ are larger than one another, $\xi _{\theta }$ has a bigger impact. Figure 20 is made to display the impact of $\xi _{z}$ in a three dimensional view. The impact of Eckert number $\xi _{ec}$ is examined in Figs. 22 and 23 on $\theta ({\eta} )$ and $\Psi ({\eta} )$. The Eckert number is the kinetic energy ratio to the fluctuation of the boundary layer in temperature used to characterize the heat dispersion. The figure shows that $\xi _{ec}$ decreases each $\theta ({\eta} )$ and $\Psi ({\eta} )$ and virtually provide the identical impact due to the increase in the kinetic energy of the fluid. Tables 5, 6, 7, 8, 9, 10, 11, 12 and 13 are made to numerically analyze the impact of various physical parameters on skin friction, magnetic flux, heat and mass fluxes. From these tables it may be observed that all findings correspond quite well with the findings of the *BVP*4*c* and HAM. The formula $\% Error = \vert \frac{N.F - H.F}{N.F}\vert$ is used to calculate the error between HAM findings (H.F) and the findings that obtained numerically (N.F) by BVP4*c*. Tables 5, 6 and 7 illustrate the torque $g'(1)$ and pressure $F_{pres}$ that the fluid exerts upon on top plate. It is noticed that increasing the distance between plates i.e $(\xi _{sq} > 0)$ reduces pressure and torque on the top plate. Elshekh et al. and Hughes et al.^5,24^ respectively found a rise in fluid pressure on the top plate, which corresponds with their experimental and theoretical findings. Similarly Tables 6 and 7 investigate the effect of $\xi _{vis}$ and $\xi _{a}$. Also Table 8 depict that an increase in MFD viscosity decrease skin friction $f''(0)$ and slightly increase heat flux $-\theta '(0)$. Similarly effect of $\xi _{mag}$ and $\xi _{ec}$ is shown in Tables 9 and 10. Tables 11 and 12 are made to show effects of $\xi _{pm}$ and $\xi _{sm}$ on $f''(0)$, $f''(0)$, $g'(0)$, $h'(0)$, $-k'({0})$ and $-\theta '(0)$.

**Table 13:** Torques on lower and upper plate with $\xi _{a} = 0.01$, $\xi _{z} = 1$, $\xi _{\theta } = 0.5$, $\xi _{mag} = 0.1$, $\xi _{pr} = \xi _{hm} = \xi _{pm} =\xi _{sm} = \xi _{ec} = 0.005$, $\xi _{vis} = S = 0.1$ and various values of $\xi _{sq}$.

$\xi _{sq}$	Rashidi et al.^15^		HAM result	
	$g'(0)$	$g'(1)$	$g'(0)$	$g'(1)$
0.1	$-\,1.08963506$	$-\,0.95987349$	$-\,1.08963672$	$-\,0.95989843$
0.2	$-\,1.17203765$	$-\,0.93844830$	$-\,1.17208879$	$-0.93846874$
0.3	$-\,1.25013649$	$-\,0.92615609$	$-\,1.25010991$	$-\,0.92612331$
0.5	$-\,1.39797797$	$-\,0.91295593$	$-\,1.39792988$	$-\,0.91296243$
1	$-\,1.73306821$	$-\,0.89280536$	$-\,1.73309746$	$-\,0.89282365$
2	$-\,2.28925762$	$-\,0.83902117$	$-\,2.28929872$	$-\,0.83903546$

## Concluding remarks

The computational model for the constitutive expressions of an unsteady Newtonian fluid is used to simulate the flow between the circular space of porous and squeezing discs in the form of equations. Using the mathematical model for the constitutive expressions of unstable Newtonian fluid, the flow between the circular space of porous and squeezing discs is described in the form of Eqs. (9–14) is subject to the boundary requirements given in Eq. (15). The series solution of the following equations is found using HAM: $f({\eta} )$, $f'({\eta} )$, $g({\eta} )$, $h({\eta} )$, $k({\eta} )$, $\theta ({\eta} )$, and $\Psi ({\eta} )$ for magnetic field components. The extraordinary stability and convergence properties of the HAM have been demonstrated. Through the use of HAM and BVP4*c*, these equations are compared for numerical investigations. Parametric analysis are carried out for the dimensionless parameters such as MFD viscosity parameter $\xi _{vis}$, magnetic Reynolds number $\xi _{mag}$, rotational Reynolds number $\xi _{a}$, squeezing Reynolds number $\xi _{sq}$, axial magnetic force parameter $\xi _{z}$, tangential magnetic force parameter $\xi _{\theta }$ and Eckert number $\xi _{ec}$. In the future, this problem could be investigated in the form of PDEs. The solution of the problem in the PDEs form will explore the physics of the problem in details.

Main upshots of this paper are presented as below:

- As the fluid’s MFD viscosity rises, the axial and azimuthal components of the magnetic field behave in opposition to one another.
- It has been discovered that a magnetic Reynolds number lowers the temperature of the fluid as well as the tangential and axial components of the velocity field.
- With an increase in the bottom plate’s rotating speed, the fluid’s torque and pressure on the plates decrease. The findings of Hughes et al.^27^ and Elshekh et al.^23^ as well as theoretical and experimental findings are in agreement with this outcome.
- For the magnetic field components, the effect of the MFD viscosity has been seen to follow a different pattern.
- The Eckert number’s effects on $\theta ({\eta} )$ and $\Psi ({\eta} )$ have been seen to be identical.
- Heat and mass transfer coefficients, as well as the distribution of the generated magnetic field, are rising functions of the rotational Reynolds number $\xi _{a}$.
- It is also observed from Table 7 that increase in fluid viscosity decrease $f''(0)$ and increase $-\theta '(0)$.

## Data Availability

The datasets used and/or analysed during the current study available from the corresponding author on reasonable request.

## List of symbols

*p*: Pressure $$({\rm N m}^{-2})$$
*x*, *y*, *z*: Cartesian coordinates (m)
$$\xi _{hm}$$: Hartmann number
*l*: Representative length between dics (m)
$H_{o}$: Uniform magnetic field
$M_{o}$: Magnetiz. for unif. magne. field
*t*: Time (s)
$T_{u}$: Fluid temperature at upper plate (K)
$T_{l}$: Fluid temperature at lower plate (K)
$C_{u}$: Fluid concentration at upper plate
$C_{l}$: Fluid concentration at lower plate
$$\xi _{pr}$$: Prandtl number$$(\nu /k)$$
$\xi _{\theta }$: Azimuthal magnetic strength
$\xi _{sq}$: Squeeze Reynolds number
$$\rho _{o}$$: Reference density
*D*(*t*): Distance between two discs (m)
$$\xi _{ec}$$: Eckert number
$C_{V,C}$: Specific heat at constant volume (J/kgK)
*M*: Magnetization
$k_{T}$: Thermal diffusion ratio
$T_{m}$: Mean fluid temperature (K)
$$K_{1}, K^{\prime}_{1}$$: Solute concentration
$$\xi _{pm}$$: Pyromagnetic coefficient
*C*: Dimensionaless concentration
$\vec{r}$: Radius vector of the disc (m)
$\xi _{rot}$: Rotational Reynolds number
$$D_{u}^{{ - 1}}$$: Representative time

## Greek symbols

$$\Omega$$: Rotation vector
*S*: Angular velocity of plates $({\rm s}^{-1})$
$\kappa$: Thermal conductivity (W/mK)
$\mu$: Dynamic viscosity of fluid (Pa s)
$\nu$: Kinematic viscosity (kg/mS)
$$\sigma$$: Electrical conductivity ($${\rm Sm}^{-1}$$)
$$\rho$$: Fluid density$$({\rm kg}/{\rm m}^3)$$
$$\alpha$$: Positive constant
$$\eta$$: Similarity variable
$\theta$: Transformed fluid temperature
$\sigma ^s$: Stefan-Boltzmann constant
$\kappa ^a$: Mean absorption co-efficient

## Subscripts

*u*: Fluid condition on upper disc
*l*: Fluid condition on lower disc

## Superscripts

$\xi _{sm}$: Salinity magnetic coefficient
$$\prime$$: Derivative w.r.t $$\eta$$
